# Novel unit B cryptophycin analogues as payloads for targeted therapy

**DOI:** 10.3762/bjoc.14.109

**Published:** 2018-06-01

**Authors:** Eduard Figueras, Adina Borbély, Mohamed Ismail, Marcel Frese, Norbert Sewald

**Affiliations:** 1 Department of Chemistry, Organic and Bioorganic Chemistry, Bielefeld University, Universitätsstraße 25, 33615 Bielefeld, Germany

**Keywords:** cryptophycin, cytotoxic agents, novel payloads, tubulin inhibitors, tumour targeting

## Abstract

Cryptophycins are naturally occurring cytotoxins with great potential for chemotherapy. Since targeted therapy provides new perspectives for treatment of cancer, new potent analogues of cytotoxic agents containing functional groups for conjugation to homing devices are required. We describe the design, synthesis and biological evaluation of three new unit B cryptophycin analogues. The *O*-methyl group of the unit B D-tyrosine analogue was replaced by an *O-*(allyloxyethyl) moiety, an *O-*(hydroxyethyl) group, or an *O-*(((azidoethoxy)ethoxy)ethoyxethyl) substituent. While the former two maintain cytotoxicity in the subnanomolar range, the attachment of the triethylene glycol spacer with a terminal azide results in a complete loss of activity. Docking studies of the novel cryptophycin analogues to β-tubulin provided a rationale for the observed cytotoxicities.

## Introduction

**Figure 1:**
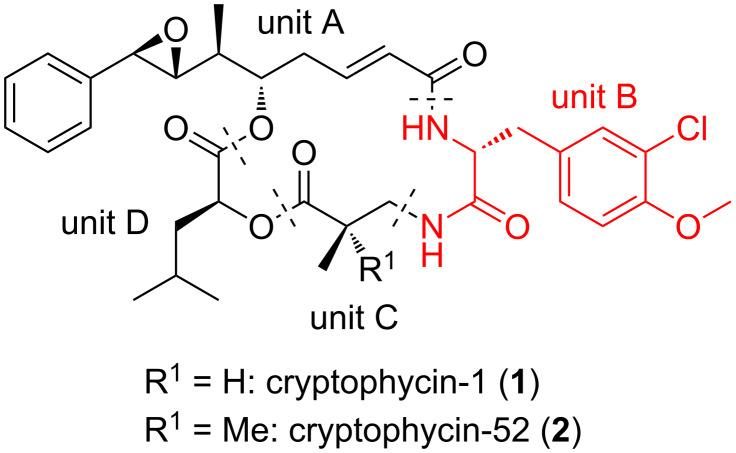
Cryptophycin-1 (**1**) and -52 (**2**).

Cryptophycins are natural occurring cyclic depsipeptides that were first isolated from cyanobacteria Nostoc sp. ATCC 53789 in 1990 [1]. Cryptophycins target tubulin, in particular the peptide site of the vinca domain. They block microtubule formation, inhibiting their assembly and, hence, are antimitotic agents [2–3]. Their high cytotoxicity prompted manifold studies that were initially focussed on the total synthesis and structure–activity relationships [4–20]. This work resulted in the identification of cryptophycin-52, a highly biologically active analogue of cryptophycin-1 (Figure 1).

Eli Lilly took cryptophycin-52 into clinical trials. Although almost half of the patients obtained a benefit from the treatment, neurotoxic side effects forced the termination of the clinical trials [21–23]. In order to overcome the side effects of cryptophycin-52 and to better understand the fundamental structure for biological activity, numerous structure–activity relationship studies have been carried out [24–35]. However, like cryptophycin-52, the new analogues were not selective against cancer cells making them not better than its parent.

In recent years the targeted delivery of cytotoxic agents has emerged as a highly promising method to tackle selectivity issues [36–40]. Cryptophycin-52 and many analogues lack an addressable group to conjugate the toxin to a homing device. For this reason, new analogues containing functional groups that would allow the conjugation of a homing device were developed [41–46]. Some of these functionalized analogues have been recently used for the preparation of antibody–drug conjugates (ADCs) and peptide–drug conjugates (PDCs) [46–51]. Nevertheless, there is still a strong need of novel cryptophycin analogues with maintained activity containing a suitable functional group that would allow the conjugation to the homing device. Cryptophycin-1 contains a methoxy group in the aromatic ring of the unit B, which is a chlorinated derivative of D-tyrosine. Different chains for unit B have been investigated, albeit the elongation of the methoxy group is still unknown. Therefore, in the current study, we embarked on the synthesis of novel cryptophycin analogues containing different substituents at the phenolic hydroxy group of the unit B. We intended to investigate whether the high biological activity of the parent compound is retained and thus, construction of ADCs and PDCs would be feasible. This preparation could be done using traceless cleavable linkers that are sensitive to the distinct physiology of the tumour with enhanced level and activity of specific enzymes. The connection between the payload and the linker is of crucial importance since its stability can dramatically change the release and thus, the activity of the compound. For this reason, the included functional groups were designed with the consideration to provide appropriate stability and activity to the future conjugate.

## Results and Discussion

### Design and synthesis

Previous docking studies have postulated that the methyl group of unit B is not involved in the cryptophycin–tubulin interaction [52]. Moreover, its absence did not produce a dramatic loss of activity [24].

Based on this, we designed cryptophycin analogues modified in the unit B. Instead of the *O-*methyl group that is present in the natural cryptophycin, we attached a hydroxyethyl group or a triethylene glycol chain terminated with an alcohol or azide, respectively. These functional groups would allow the conjugation of the novel cryptophycin analogues across an appropriate linker to an antibody or peptide. Either a virtually uncleavable triazole (introduced by CuAAC) or scissile ester, carbonate, or carbamate moieties were taken into account.

**Scheme 1:**
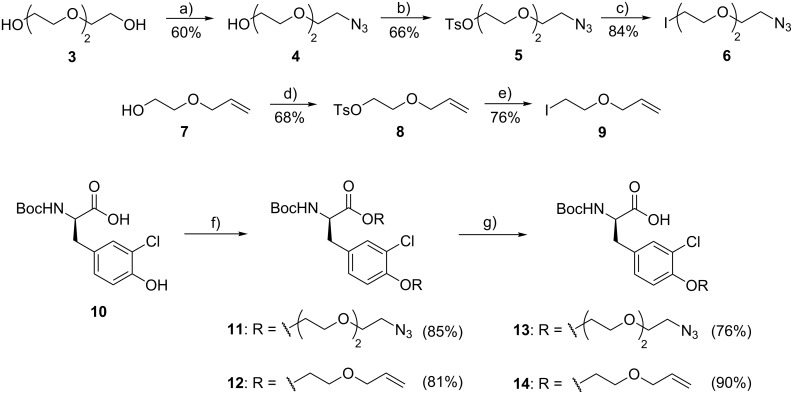
Synthesis of modified unit B (**13** and **14**). Reagents and conditions: (a) 1) TsCl, DMAP, Et_3_N, CH_2_Cl_2_, rt, 4 h; 2) NaN_3_, DMF, 70 °C, overnight; (b) TsCl, Et_3_N, CH_2_Cl_2_, rt, overnight; (c) NaI, acetone, reflux, overnight; (d) TsCl, Et_3_N, CH_2_Cl_2_, rt, overnight; (e) NaI, acetone, reflux, overnight; (f) **6** or **9**, K_2_CO_3_, DMF, 50 °C, overnight; (g) LiOH, H_2_O/MeOH/THF 1:1:1, rt, 2 h.

The synthesis of the modified unit B (Scheme 1) started with the preparation of the two different spacers that were later connected to the phenol. Starting from triethylene glycol (**3**) or 2-allyloxyethanol (**7**) tosylations and nucleophilic displacements by azide or iodide substitution provided **6** and **9** in good yields. O*-*Alkylation of Boc-protected 3-chlorinated D-tyrosine **10** with **6** or **9** gave **11** and **12**, again in satisfactory yields (81–85%). Saponification of the ester moiety in **11** and **12** that was formed concomitantly with the O*-*alkylation in the previous reaction provided Boc-protected modified units B **13** and **14** in 76 and 90% yield, respectively.

**Scheme 2:**
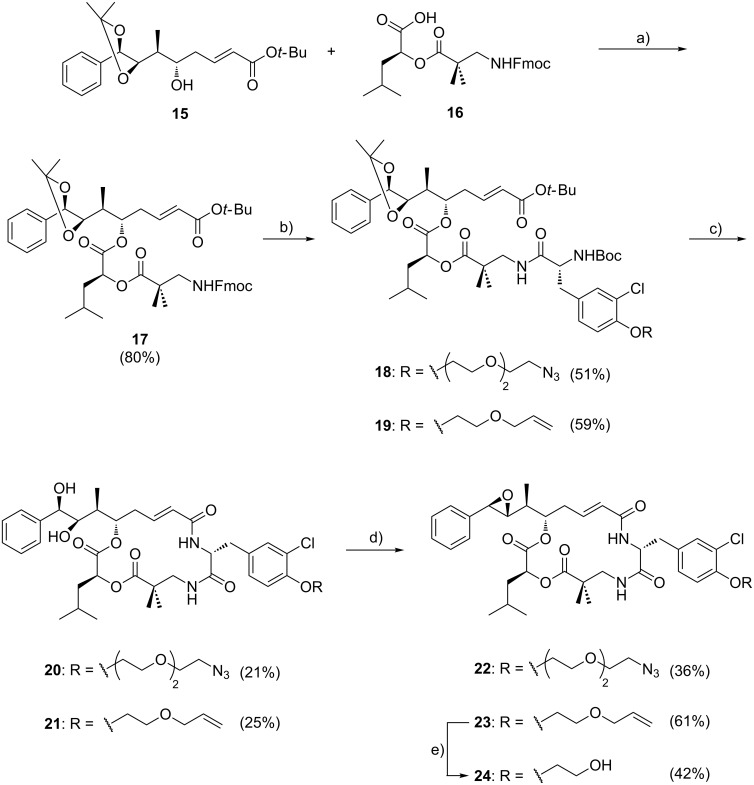
Synthesis of cryptophycin analogues **22**, **23** and **24**. Reagents and conditions: (a) 4-DMAP, 2,4,6-trichlorobenzoyl chloride, Et_3_N, THF, 0 °C, 3 h; (b) 1) piperidine, DMF, rt, 2 h; 2) **13** or **14**, HOAt, EDC·HCl, Et_3_N, CH_2_Cl_2_, 0 °C → rt, overnight; (c) 1) TFA/CH_2_Cl_2_/H_2_O, rt, 2 h; 2) HATU, HOAt, DIPEA, DMF, rt, slow addition + 2 h; (d) 1) (CH_3_O)_3_CH, PPTS, CH_2_Cl_2_, rt, 2 h; 2) AcBr, CH_2_Cl_2_, rt, 4 h; 3) K_2_CO_3_, DME/ethylene glycol (2:1 v/v), rt, 5 min; (e) Pd(PPh_3_)_4_, phenylsilane, CH_2_Cl_2_, rt, 7 h.

The synthesis of units C–D and A succeeded as previously described in the literature; unit A (**15**) and C–D (**16**) were connected by Yamaguchi esterification to give **17** (Scheme 2) [45]. Then, Fmoc was cleaved from the N-terminus of unit C–D–A (**17**) using piperidine and the resulting crude amine was coupled to the corresponding modified unit B (**13** or **14**), affording the according linear cryptophycins **18** and **19** in acceptable yields (51–59%). Compounds **18** and **19** were treated with trifluoroacetic acid for simultaneous Boc and *t*-Bu removal, which also cleaved the dioxolane ring. Subsequently, macrolactamization was performed under pseudo-high-dilution conditions to afford **20** and **21** as described previously [16]. Then the diol was transformed into the epoxide following a three-step one-pot reaction as extensively used in the synthesis of cryptophycin analogues [46]. Cryptophycin analogues **22** and **23** were obtained in good purity after column chromatography. The allyl ether in **23** was cleaved using Pd(PPh_3_)_4_ as Pd(0) source and phenylsilane as scavenger to obtain the cryptophycin analogue **24** in good purity.

### Biological evaluation

The biological activity of the modified unit B analogues was determined in a cell viability assay using the human cervix carcinoma cell line KB-3-1 (Table 1). The cryptophycin analogue **22** showed a dramatic loss of activity compared to cryptophycin-52 (**2**), while analogues **23** and **24** showed a reduced cytotoxicity although their IC_50_ values are still in the low nanomolar range. The observed dramatic loss of activity of analogue **22** could be due to its poor internalization or the modification could alter the interaction with tubulin. In order to get an extensive knowledge of the novel analogues, we embarked in docking and modelling studies, herein reported, and internalization studies are ongoing in our research group.

**Table 1:** Cytotoxicity of cryptophycin-52 and its unit B analogues.

compd	unit B	IC_50_ KB-3-1 (nM)

**2**	CH_2_Ph(*m*-Cl,*p*-OMe)	0.015
**22**	CH_2_Ph(*m*-Cl,*p*-(OCH_2_CH_2_)_3_N_3_)	195000
**23**	CH_2_Ph(*m*-Cl,*p*-OCH_2_CH_2_OCH_2_CHCH_2_)	0.748
**24**	CH_2_Ph(*m*-Cl,*p*-OCH_2_CH_2_OH)	0.184

### Docking and modelling of cryptophycin derivatives

**Figure 2:**
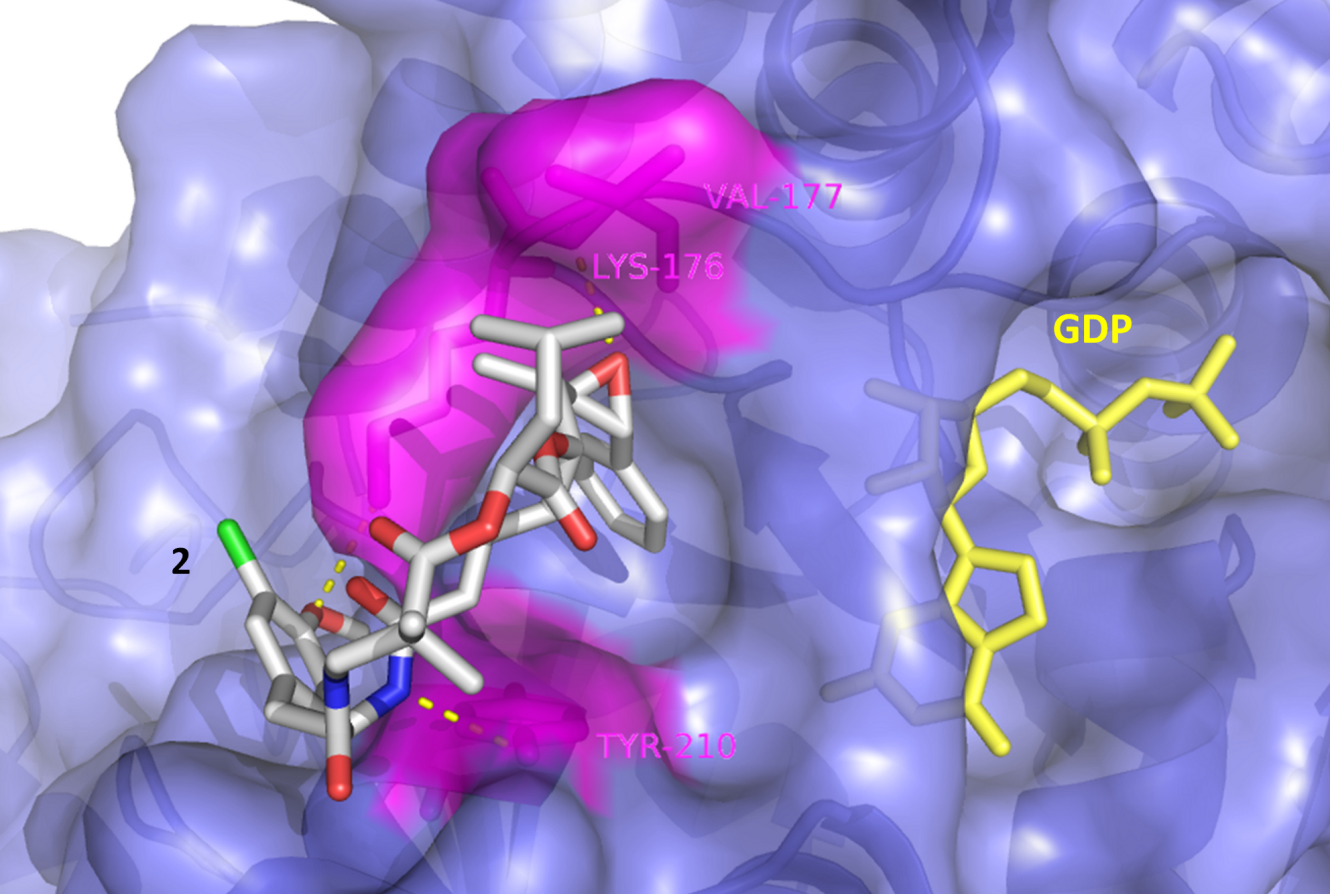
Binding mode of **2**, showing the interaction to the vinca domain peptide binding pocket (blue). Hydrogen bonds are shown as yellow dots with the interacting amino acid residues in magenta.

There is no X-ray analysis of cryptophycin–tubulin complexes available to provide information on the binding site. Based on biochemical evidence, binding close to the vinca-alkaloid binding site of β-tubulin, the so called “peptide-site”, has been proposed [2,52–53]. We performed a docking study to explain the different affinities of the newly synthesized derivatives. The parent compound **2** scored highest with respect to β-tubulin binding (Table 2). Three hydrogen bonds were detected to key residues in the peptide binding pocket of the vinca domain (Lys176, Val177 and Tyr210). Other than previously reported [52], the methoxy group of subunit B forms a hydrogen bond with Lys176 (Figure 2). Another binding mode of **2** with high binding affinity and hydrogen bond formation did not involve any interaction of subunit B, yet it was oriented towards the GDP binding site that might influence GTP hydrolysis.

**Table 2:** Binding energies for the different cryptophycin analogues.

compd	binding energy (kJ/mol)	max. binding energy (kJ/mol)	min. binding energy (kJ/mol)

**2**	36.17	36.17	17.21
**22**	22.61	22.61	5.44
**23**	32.20	32.20	10.38
**24**	32.70	32.70	11.72

**Figure 3:**
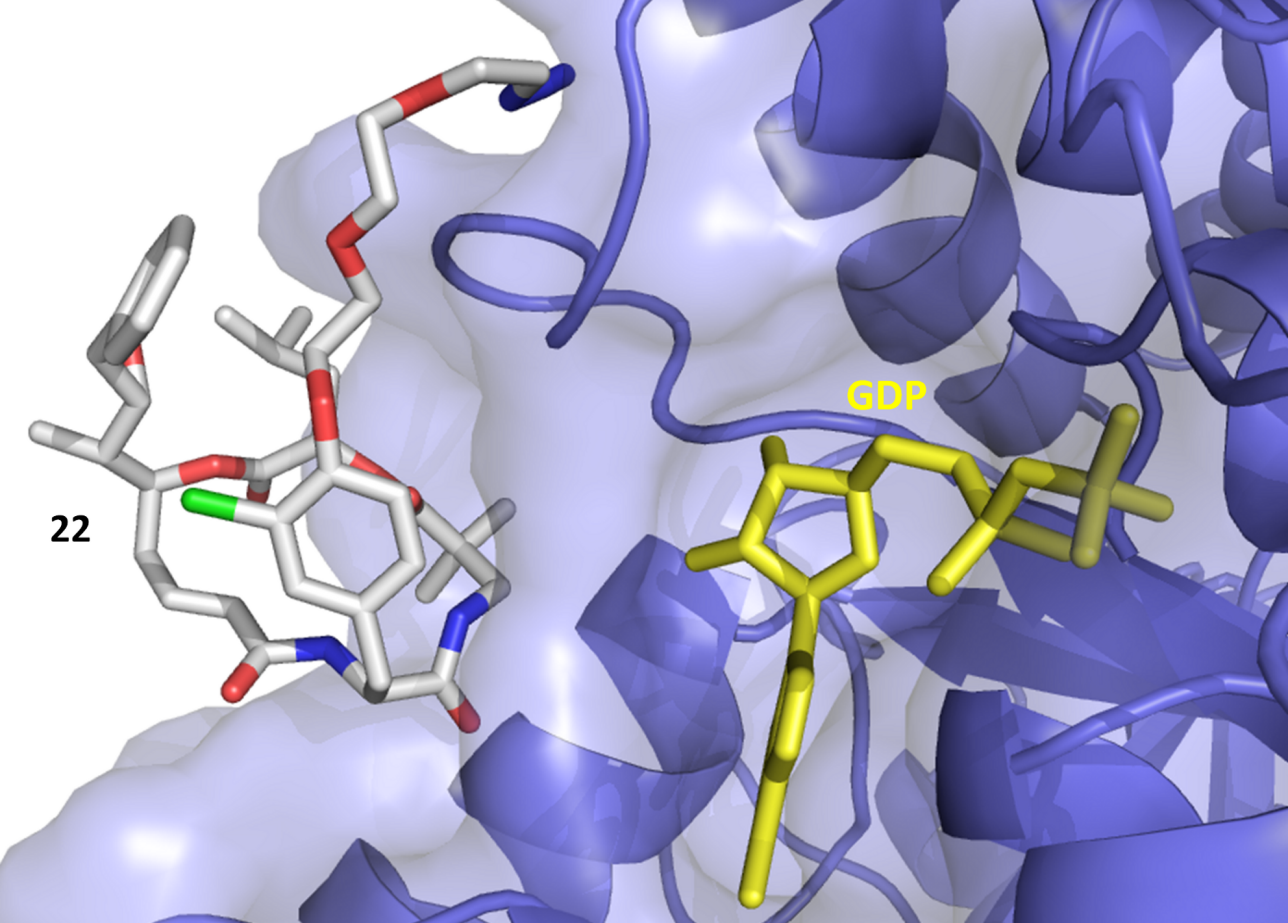
Docking of **22** to the vinca domain of β-tubulin. Surface and backbone of β-tubulin are shown in blue, GDP in yellow. No hydrogen bond formation was detected. The orientation of the azidoethoxy-ethoxyethyl substituent prevents the inhibitor from the correct interaction with the protein. The epoxide and benzyl group of subunit A are pointing away from the binding pocket.

**Figure 4:**
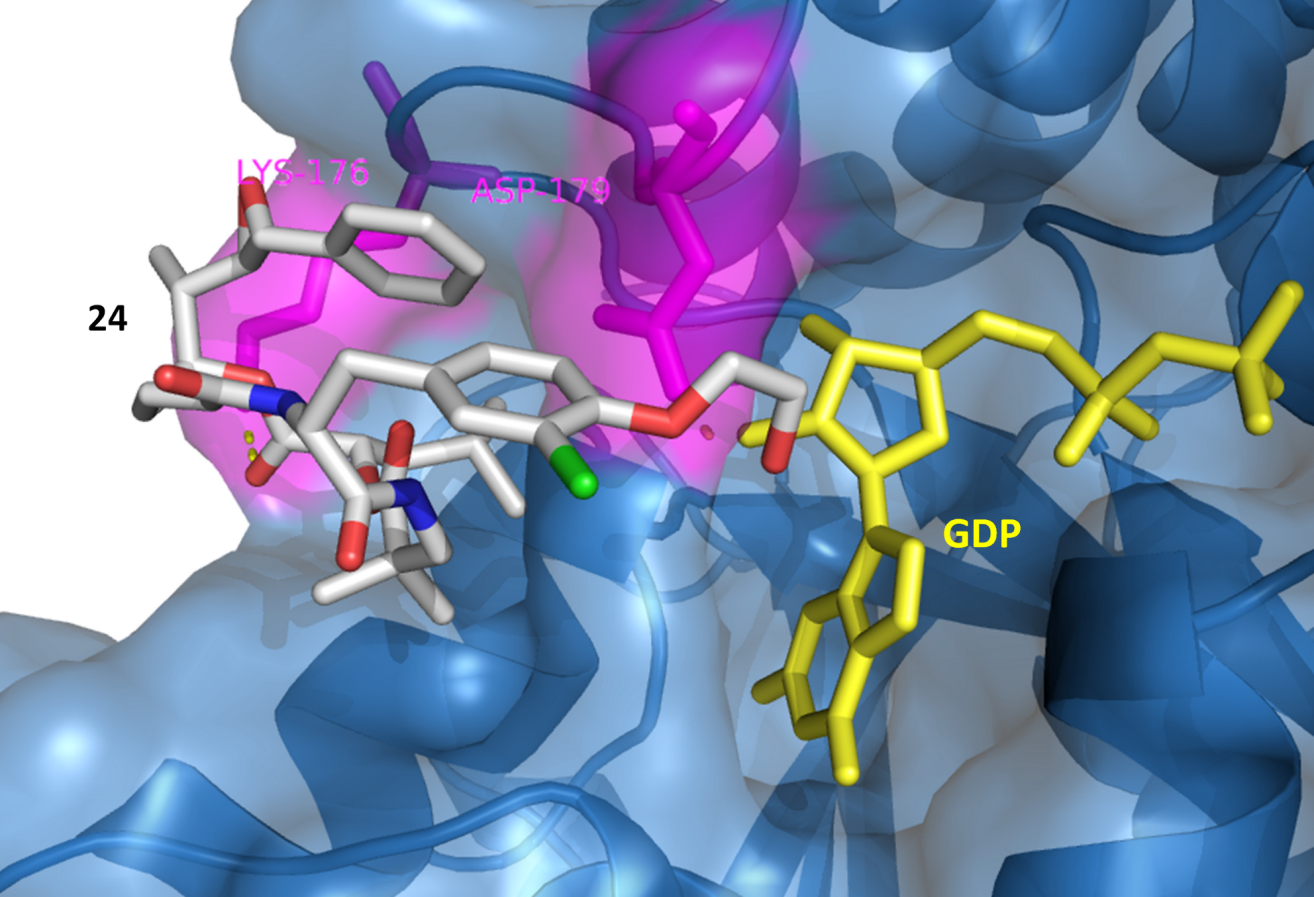
Docking of **24** to β-tubulin. Surface and backbone of β-tubulin are shown in blue, GDP in yellow. H-bonding (yellow dots) was detected with Lys176 and Asp179 in magenta. The benzyl group and the epoxide of subunit A are directed towards the peptide binding pocket, while the hydroxyethyl group is positioned towards the GDP binding pocket forming an H-bond with Asp179.

Compound **22** with the triethylene glycol-based substituent prevents correct binding, the binding energy was decreased and mainly nonspecific interactions outside the binding pocket were observed (Figure 3). This was not the case for the other derivatives **23** and **24** (Figure 4).

Besides hydrogen bond formation and binding affinity of inhibitors **2**, **23** and **24**, π-interactions and hydrophobic contacts with the binding pocket of the vinca domain were detected that would in turn increase the affinity of the inhibitor and its effect on the protein (Supporting Information File 1, Table S1).

## Conclusion

In summary, three new cryptophycin analogues with a modified unit B have been designed and successfully synthesized. The novel analogues were less active than cryptophycin-52 in the KB-3-1 cell line. Analogue **22** showed a dramatic loss of activity whereas analogues **23** and **24** showed a reduced activity but were still very cytotoxic.

## Supporting Information

File 1 Experimental part and analytical data.
