# Supplementary material for: Novel unit B cryptophycin analogues as payloads for targeted therapy
Source: Beilstein J Org Chem. 2018 Jun 1;14:1281–6. doi: 10.3762/bjoc.14.109 (PMC6009196; doi:10.3762/bjoc.14.109)
Supplement: File 1 — Experimental part and analytical data. [file Beilstein_J_Org_Chem-14-1281-s001.pdf]

Supporting Information

for

Novel unit B cryptophycin analogues as payloads for targeted therapy

Eduard Figueras, Adina Borbély, Mohamed Ismail, Marcel Frese and Norbert Sewald*

Address: Department of Chemistry, Organic and Bioorganic Chemistry, Bielefeld University, Universitätsstraße 25, 33615 Bielefeld, Germany

Email: Norbert Sewald - Norbert.sewald@uni-bielefeld.de

*Corresponding author

Experimental part and analytical data

Table of contents

1) General methods S2

2) Synthesis of modified unit B S5

3) Synthesis of new cryptophycins S10

4) NMR, HPLC and mass spectra S19

5) References S40

1) General methods

All reactions requiring anhydrous conditions were performed under argon atmosphere. DMF was dried over 4 Å molecular sieves, CH_2Cl_2 was distilled from CaH_2, THF and DME were distilled from sodium/benzophenone. Anhydrous acetone and ethylene glycol were purchased from commercial sources. All the other chemicals and solvents (HPLC-grade or reagent-grade quality), unless otherwise stated, were purchased from commercial sources and used without further purification. Silica for flash chromatography was purchased from Macherey-Nagel 40–63 µM (230-400 mesh). Reactions were monitored by thin layer chromatography using aluminium-backed plates coated with silica gel 60 F254 from Merck; visualization was accomplished with UV light or staining with potassium permanganate or cerium molybdate solution.

Liquid chromatography–mass spectrometry

LC–MS was conducted using an Agilent 1200 series consisting of an autosampler, degasser, binary pump, column oven and diode array detector coupled to an Agilent 6220 accurate-mass TOF MS. A Hypersil Gold C_{18} (150 mm × 2.1 mm, 3 µm particle size) was used as column.

Eluent A: $H_2O/CH_3CN/HCOOH$ = 95/5/0.1 and eluent B: $H_2O/CH_3CN/HCOOH$ = 5/95/0.1.

Method A:

Flow rate: 300 µl/min

0 min	100% A	0% B
10 min	2% A	98% B
11 min	2% A	98% B
11.5 min	100% A	0% B
15 min	100% A	0% B

High-resolution mass spectrometry

High resolution mass spectra were recorded on an Agilent 6220 accurate-mass TOF LC/MS. Samples were injected through an Agilent 1200 series. Hypersil Gold C_{18} (50 mm × 2.1 mm, 1.9 µm particle size) was used as a column. Same solvents than HPLC–MS were used and a linear gradient from 0 to 98% B over 4 minutes was employed.

The mass spectrometer was externally calibrated using Agilent tuning mix prior to measurement.

NMR spectroscopy

NMR spectra were recorded on a Bruker Avance 400 (^{1}H: 400 MHz, ^{13}C: 100 MHz), Avance 500HD (^{1}H: 500 MHz, ^{13}C: 126 MHz) or Avance 600 (^{1}H: 600 MHz, ^{13}C: 151 MHz) at 298 K. Chemical shifts were referenced to residual nondeuterated solvent signal ($CDCl_3$: ^{1}H: 7.26 ppm; ^{13}C: 77.16 ppm). Coupling constants (*J*) are reported in Hz with the following abbreviations used to indicate splitting: s = singlet, d = doublet, t = triplet, q = quartet, m = multiplet, br = broad signal. The acronyms uA, uB, uC and uD describe signals pertaining to cryptophycin units A–D.

Cell lines

Biological tests: The KB-3-1 cells were cultivated as monolayer in DMEM (Dulbecco's modified Eagle medium) with glucose (4.5 g/L), L-glutamine, sodium pyruvate, phenol red (PAA) and supplemented with 10% foetal calf serum (FCS). The cells were maintained at 37 °C and 5.3% CO_2/humidified air. On the day before the test, the cells were detached with trypsin/ethylenediaminetetraacetic acid (EDTA) solution (0.05% / 0.02% in phosphate buffered saline solution PBS; PAA) and plated in sterile 96-well plates in a density of 10 000 cells in 100 µL medium per well. The dilution series of the compounds were prepared from stock solutions in DMSO of concentrations of 1 mM or 10 mM. The stock solutions were diluted with culture medium (10% FCS). The dilution (100 µL) was added to the wells. Each concentration was tested in six replicates. The control contained the same concentration of DMSO as the first dilution. After incubation for 72 h at 37 °C and 5.3% CO_2/humidified air, 30 µL of an aqueous resazurin solution (175 µM) was added to each well. Again, the cells were incubated at the same conditions for 6 h. Then the fluorescence was measured using a TECAN infinite M200. The excitation was effected at a wavelength of 530 nm, whereas the emission was recorded at a wavelength of 588 nm. The IC_{50} values were calculated as a sigmoidal dose response curve using GraphPad Prism (version 4.03). The IC_{50} values equal the drug concentrations, at which vitality is 50%.

Docking and molecular dynamic simulation

The αβ-tubulin structure was obtained from protein data bank (pdb: 1jff). The α-subunit of the protein was removed, the β-subunit was modified by adding the missing hydrogens and

the ligand (taxol) was removed. Structures of the different tested cryptophycin derivatives were built by Yasara structure with the correct stereochemistry. Both the beta subunit of tubulin and cryptophycin derivatives were energy minimized using the Yasara2 force field before using in further experiments.

Docking was performed by Yasara structure using Autodock [1], the simulation cell was defined around the vinca domain residues of the energy minimized beta subunit for docking of all cryptophycin derivatives. Docking results were analysed based on the B-factor (binding energy) calculated by Yasara, compounds with correct positioning and high binding energies were used for further analysis.

Molecular dynamic simulation was done by Yasara structure; the selected docking modes were used for simulation. Simulation cell was extended 10 Å around the whole structure and filled with water of 0.99 g ml^{-1} density and randomized molecule orientation, AMBER15IPQ with its default parameters was used as force field for running the simulation up to 1 ns. Simulation was also performed on the Apo structure for comparative analysis using same parameters.

Table S1: Interaction of the cryptophycin derivatives with key amino acid residues within the vinca domain.

Inhibitor	Subunit*	Hydrophobic contact	Pi interaction
**2**	A	S174, Y210, V177, Y224	Y210
	B	Y210, E207, D211	Y210
**22**	C	K176	–
	D	V177	
**23**	A	K176, S174, V177, E207	Y210
	B	S174, Y224, V177, D179	–
**24**	A	V177, S178	–
	C	P222, Y224	
	D	E207, Y224	

* subunit of cryptophycin molecule.

2) Synthesis of modified unit B

2-(2-(2-Azidoethoxy)ethoxy)ethan-1-ol (4)

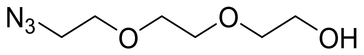

Triethylene glycol (10 g, 66.6 mmol, 1 equiv) was monotosylated as previously described and used without column chromatography purification [2]. The crude was dissolved in anhydrous DMF (28 mL) under inert atmosphere and NaN_3 (1.83 g, 28.2 mmol, 2 equiv) was added. The solution was stirred overnight at 70 ºC. Then, the DMF was removed under reduced pressure and the residue was dissolved in DCM (100 mL) and HCl 0.5 M (100 mL), the layers were separated and the aqueous layer was further extracted with DCM (100 mL). The combined organic layers were dried over Na_2SO_4 and the solvent was removed under reduced pressure. The crude was purified by column chromatography using DCM/MeOH (99:1 → 96:4) as eluent to afford 1.75 g (60% yield) of **4** as a colourless oil.

^{1}H-NMR (400 MHz, $CDCl_3$): δ (ppm) = 3.40 (t, *J* = 5.0 Hz, 2H, CH_2N_3), 3.62 (m, 2H, $C\underline{H}_2CH_2OH$), 3.67-3.70 (m, 6H, CH_2), 3.75 (m, 2H, $C\underline{H}_2OH$).

^{13}C-NMR (100 MHz, $CDCl_3$): δ (ppm) = 50.7, 61.8, 70.1, 70.4, 70.7, 72.5.

2-(2-(2-Azidoethoxy)ethoxy)ethyl 4-methylbenzenesulfonate (5)

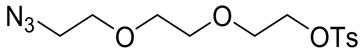

2-(2-(2-Azidoethoxy)ethoxy)ethan-1-ol (1.67 g, 9.53 mmol, 1 equiv) was monotosylated as previously described and the crude was purified by column chromatography using hexane/ethyl acetate (7:3) as eluent to obtain 2.07 g (66% yield) of **5** as colourless oil.

^{1}H-NMR (400 MHz, $CDCl_3$): δ (ppm) = 2.44 (s, 3H, CH_3), 3.36 (t, *J* = 5.1 Hz, 2H, CH_2N_3), 3.60 (s, 4H, CH_2), 3.64 (m, 2H, CH_2), 3.70 (m, 2H, $C\underline{H}_2CH_2OSO_2$), 4.16 (m, 2H, CH_2OS_2), 7.34 (d, *J* = 8.0 Hz, 2H, $C^{ar}H$), 7.79 (d, *J* = 8.3 Hz, 2H, $SO_2C^{ar}C^{ar}H$).

^{13}C-NMR (100 MHz, $CDCl_3$): δ (ppm) = 21.8, 50.8, 68.9, 69.4, 70.2, 70.7, 70.9, 128.1, 129.9, 133.1, 144.9.

1-Azido-2-(2-(2-iodoethoxy)ethoxy)ethane (6)

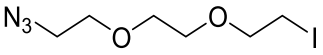

2-(2-(2-Azidoethoxy)ethoxy)ethyl 4-methylbenzenesulfonate (1.2 g, 3.64 mmol, 1 equiv) and NaI (2.18 g, 14.64 mmol, 4 equiv) were dissolved in anhydrous acetone (28 mL) under inert atmosphere and it was refluxed overnight. Then, the solvent was removed under reduced pressure and the crude was dissolved in DCM (100 mL), washed with H_2O (100 mL) and brine (100 mL). The organic layer was dried over Na_2SO_4 and the solvent was removed under reduced pressure to obtain 0.87 g (84% yield) of **6** as colourless oil.

^{1}H-NMR (400 MHz, $CDCl_3$): δ (ppm) = 3.27 (t, *J* = 6.8 Hz, 2H, CH_2I), 3.40 (t, *J* = 5.0 Hz, 2H, CH_2N_3), 3.68-3.71 (m, 6H, CH_2), 3.77 (t, *J* = 6.8 Hz, 2H, $C\underline{H_2}CH_2I$).

2-(Allyloxy)ethyl 4-methylbenzenesulfonate (8)

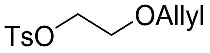

2-Allyloxyethanol (5 g, 48.9 mmol, 1 equiv) was tosylated as previously described and the crude product was purified by column chromatography using PE/EtOAC (8:2) as eluent to obtain 8.48 g (68% yield) of **8** as colourless oil.

^{1}H-NMR (500 MHz, $CDCl_3$): δ (ppm) = 2.44 (s, 3H, CH_3), 3.62 (t, *J* = 4.8 Hz, 2H, $C\underline{H_2}OAllyl$), 3.93 (d, *J* = 5.6 Hz, 2H, $OC\underline{H_2}CH$), 4.16 (t, *J* = 4.8 Hz, 2H, CH_2OSO_2), 5.15 (dd, *J* = 10.6, 1.5 Hz, 1H, $CH{=}C\underline{H^{cis}}H^{trans}$), 5.21 (dd, *J* = 17.3, 1.5 Hz, 1H, $CH{=}CH^{cis}\underline{H^{trans}}$), 5.81 (ddt, *J* = 16.3, 10.8, 5.6 Hz, 1H, $C\underline{H}{=}CH_2$), 7.33 (d, *J* = 8.0 Hz, 1H, CH^{ar}), 7.79 (d, *J* = 8.0 Hz, 1H, CH^{ar}).

^{13}C-NMR (126 MHz, $CDCl_3$): δ (ppm) = 21.7, 67.5, 69.4, 72.2, 117.5, 128.1, 129.9, 133.1, 134.2, 144.9.

3-(2-Iodoethoxy)prop-1-ene (9)

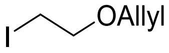

2-(Allyloxy)ethyl 4-methylbenzenesulfonate (8.45 g, 33.0 mmol, 1 equiv) and NaI (19.8 g, 131.9 mmol, 4 equiv) were dissolved in anhydrous acetone (65 mL) under inert atmosphere and it was refluxed overnight. Then, the solvent was removed under reduced pressure and the

crude was dissolved in DCM (150 mL), washed with H_2O (150 mL) and brine (150 mL). The organic layer was dried over Na_2SO_4 and the solvent was removed under reduced pressure to obtain 5.32 g (76% yield) of **9** as colourless oil.

^{1}H-NMR (500 MHz, $CDCl_3$): δ (ppm) = 3.26 (t, *J* = 6.8 Hz, 2H, CH_2I), 3.70 (t, *J* = 6.8 Hz, 2H, $C\underline{H_2}CH_2I$), 4.04 (d, *J* = 5.6 Hz, 2H, $OC\underline{H_2}CH$), 5.21 (d, *J* = 10.4 Hz, 1H, $CH{=}C\underline{H^{cis}}H^{trans}$), 5.30 (dd, *J* = 17.3, 1.5 Hz, 1H, $CH{=}CH^{cis}\underline{H^{trans}}$), 5.91 (ddt, *J* = 16.7, 11.1, 5.6 Hz, 1H, $C\underline{H}{=}CH_2$).

^{13}C-NMR (126 MHz, $CDCl_3$): δ (ppm) = 3.0, 70.8, 71.9, 117.7, 134.5.

Boc-D-Tyr(3-Cl,$(OCH_2CH_2)_3N_3$)-$(OCH_2CH_2)_3N_3$ (11)

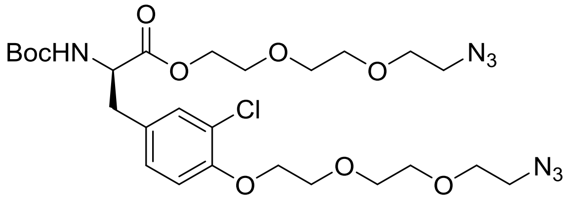

Boc-D-Tyr(3-Cl)-OH (1.1 g, 3.49 mmol, 1 equiv), K_2CO_3 (2.12 g, 15.35 mmol, 4.4 equiv) and **6** (2.2 g, 7.72 mmol, 2.2 equiv) were placed under argon atmosphere, dissolved with anhydrous DMF (22 mL) and the solution was stirred overnight at 50 ºC. Then, it was diluted with EtOAc (150 mL) and H_2O (150 mL), the layers were separated and the aqueous layer was further extracted with EtOAc (150 mL). The combined organic layers were washed with brine (200 mL), dried over Na_2SO_4 and the solvent was removed under reduced pressure. The crude was purified by column chromatography using PE/EtOAC (1:1) as eluent to obtain 1.88 g (85% yield) of **11** as colourless oil.

^{1}H-NMR (400 MHz, $CDCl_3$): δ (ppm) = 1.43 (s, 9H, $(CH_3)_3C$), 2.99 (dd, *J* = 14.0, 5.7 Hz, 1H, CH_2-β), 3.07 (dd, *J* = 14.0, 5.7 Hz, 1H, CH_2-β), 3.38 (m, 4H, CH_2N_3), 3.65-3.72 (m, 12H, OCH_2CH_2O), 3.77-3.80 (m, 2H, OCH_2), 3.90 (t, *J* = 4.9 Hz, 2H, $C^{ar}OCH_2C\underline{H_2}$), 4.16 (t, *J* = 4.9 Hz, 2H, $C^{ar}OC\underline{H_2}CH_2$), 4.23-4.34 (m, 2H, OCH_2), 4.55 (q, *J* = 6.3 Hz, 1H, $C^{\alpha}H$), 5.01 (d, *J* = 8.1 Hz, NH, 1H), 6.86 (d, *J* = 8.4 Hz, 1H, $C^{5'}H$), 6.99 (dd, *J* = 8.4, 2.2 Hz, 1H, $C^{6'}H$), 7.16 (d, *J* = 2.2 Hz, 1H, $C^{2'}H$).

^{13}C-NMR (100 MHz, $CDCl_3$): δ (ppm) = 28.4, 37.2, 50.8, 50.8, 54.4, 64.6, 69.0, 69.1, 69.7, 70.2, 70.2, 70.8, 70.8, 70.9, 71.2, 80.1, 113.8, 123.0, 128.7, 129.6, 131.3, 153.5, 155.1, 171.7.

Boc-D-Tyr(3-Cl,$(OCH_2CH_2)_3N_3$)-OH (13)

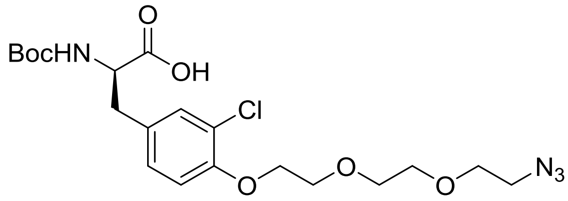

11 (0.72 g, 1.14 mmol, 1 equiv) was dissolved in MeOH/THF (1:1, 6 mL total) and Li-OH·H_2O (72 mg, 1.71 mmol, 1.5 equiv) in H_2O (3 mL) was added dropwise and the solution was stirred for 2 h at rt. Then, H_2O (30 mL) was added and the pH was adjusted to 3 with 1M $KHSO_4$. The solution was extracted with EtOAc (3 x 30 mL) and the combined organic layers were thoroughly washed with 1 M HCl (3 × 50 mL) and brine (50 mL). The organic layer was dried over Na_2SO_4 and the solvent was removed under reduced pressure to obtain 0.41 g (76% yield) of **13** as white solid.

^{1}H-NMR (400 MHz, $CDCl_3$, rotamers): δ (ppm) = 1.34/1.43 (2 s, 9H, $(CH_3)_3C$), 2.85 (m, 0.3H, $C^\beta\underline{H^A}H^B$), 2.98 (dd, J = 14.4, 6.3 Hz, 0.7H, $C^\beta\underline{H^A}H^B$), 3.11 (dd, J = 14.4, 5.4 Hz, 1H, $C^\beta H^A\underline{H^B}$), 3.38 (t, J = 5.1 Hz, 2H, CH_2N_3), 3.67-3.70 (m, 4H, OCH_2), 3.78-3.80 (m, 2H, OCH_2), 3.91 (t, J = 5.1 Hz , 2H, $C^{ar}OCH_2C\underline{H_2}$), 4.16 (t, J = 5.1 Hz , 2H, $C^{ar}OCH_2$), 4.35 (m, 0.3H, $C^\alpha H$), 4.53 (q, J = 6.5 Hz, 0.7H, $C^\alpha H$), 5.00 (d, J = 7.5 Hz, 0.7H, NH), 6.28 (br, 0.3H, NH), 6.87 (d, J = 8.4 Hz, 1H, $C^{5'}H$), 7.02 (dd, J = 8.4, 2.2 Hz, 1H, $C^{6'}H$), 7.19 (br m, 1H, $C^{2'}H$).

^{13}C-NMR (100 MHz, $CDCl_3$): δ (ppm) = 28.4, 36.8, 50.8, 54.4, 69.1, 69.7, 70.2, 70.9, 71.2, 80.6, 113.9, 123.1, 128.7, 129.5, 131.3, 153.6, 155.5, 175.4.

Boc-D-Tyr(3-Cl,CH_2CH_2OAllyl)-OCH_2CH_2OAllyl (12)

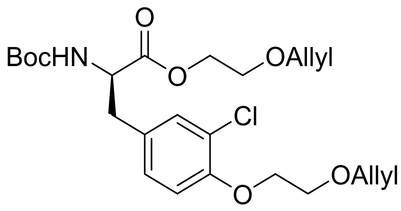

Boc-D-Tyr(3-Cl)-OH (1 g, 3.16 mmol, 1 equiv), K_2CO_3 (1.92 g, 13.90 mmol, 4.4 equiv) and **9** (1.47 g, 6.95 mmol, 2.2 equiv) were placed under argon atmosphere, dissolved with anhydrous DMF (20 mL) and the solution was stirred overnight at 50 °C. Then, it was diluted with EtOAc (150 mL) and H_2O (150 mL), the layers were separated and the aqueous layer was

further extracted with EtOAc (150 mL). The combined organic layers were washed with brine (200 mL), dried over Na_2SO_4 and the solvent was removed under reduced pressure. The crude was purified by column chromatography using PE/EtOAC (4:1) as eluent to obtain 1.24 g (81% yield) of **12** as colourless oil.

^{1}H-NMR (500 MHz, $CDCl_3$): δ (ppm) = 1.42 (s, 9H, $(CH_3)_3C$), 2.99 (dd, J = 14.2, 5.9 Hz, 1H, CH_2-β), 3.07 (dd, J = 14.2, 5.9 Hz, 1H, CH_2-β), 3.59-3.66 (m, 2H, $COOCH_2C\underline{H_2}$), 3.84 (t, J = 4.9 Hz, 2H, $C^{ar}OCH_2C\underline{H_2}$), 4.02 (d, J = 5.6 Hz, 2H, $COO(CH_2)_2OC\underline{H_2}$), 4.13 (d, J = 5.6 Hz, $C^{ar}O(CH_2)_2OC\underline{H_2}$), 4.16 (t, J = 4.9 Hz, 2H, $C^{ar}OC\underline{H_2}$), 4.22-4.26 (m, 1H, $COOC\underline{H^A}H^B$), 4.30-4.34 (m, 1H, $COOCH^A\underline{H^B}$), 4.55 (q, J = 6.4 Hz, 1H, $C^{\alpha}H$), 5.00 (d, J = 8.2 Hz, NH, 1H), 5.20 (d, J = 10.4 Hz, 2H, $CH{=}C\underline{H^{cis}}H^{trans}$), 5.28 (d, J = 16.3 Hz, 1H, $CH{=}CH^{cis}\underline{H^{trans}}$), 5.32 (d, J = 16.3 Hz, 1H, $CH{=}CH^{cis}\underline{H^{trans}}$), 5.86-5.97 (m, 2H, $C\underline{H}{=}CH_2$), 6.86 (d, J = 8.3 Hz, 1H, $C^{5'}H$), 6.99 (dd, J = 8.3, 2.2 Hz, 1H, $C^{6'}H$), 7.15 (d, J = 2.2 Hz, 1H, $C^{2'}H$).

^{13}C-NMR (126 MHz, $CDCl_3$): δ (ppm) = 28.4, 37.2, 54.5, 64.6, 67.7, 68.4, 69.1, 72.2, 72.6, 80.1, 113.9, 117.3, 117.6, 123.1, 128.7, 129.7, 131.4, 134.4, 134.7, 153.6, 155.1, 171.7.

Boc-D-Tyr(3-Cl,CH_2CH_2OAllyl)-OH (14)

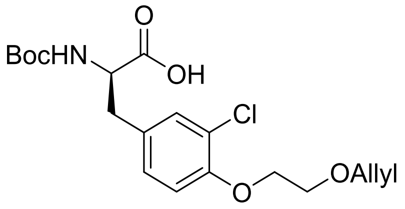

12 (1.23 g, 2.55 mmol, 1 equiv) was dissolved in MeOH/THF (1:1, 13 mL total) and LiOH·H_2O (0.16 g, 3.83 mmol, 1.5 equiv) in H_2O (6.5 mL) was added dropwise and the solution was stirred for 2 h at rt. Then, H_2O (50 mL) was added and the pH was adjusted to 3 with 1M $KHSO_4$. The solution was extracted with EtOAc (3 × 50 mL) and the combined organic layers were thoroughly washed with 1 M HCl (3 × 50 mL) and brine (50 mL). The organic layer was dried over Na_2SO_4 and the solvent was removed under reduced pressure to obtain 0.92 g (90% yield) of **14** as white solid.

^{1}H-NMR (500 MHz, $CDCl_3$, rotamers): δ (ppm) = 1.33/1.42 (2 s, 9H, $(CH_3)_3C$), 2.81-2.85 (m, 0.3H, $C^{\beta}\underline{H^A}H^B$), 2.99 (dd, J = 14.0, 6.4 Hz, 0.7H, $C^{\beta}\underline{H^A}H^B$), 3.12 (dd, J = 14.2, 5.6 Hz, 1H, $C^{\beta}H^A\underline{H^B}$), 3.85 (t, J = 4.9 Hz, 2H, $C^{ar}OCH_2C\underline{H_2}$), 4.13 (d, J = 5.6 Hz, $C^{ar}O(CH_2)_2OC\underline{H_2}$), 4.16 (t, J = 4.9 Hz, 2H, $C^{ar}OC\underline{H_2}$), 4.34 (m, 0.3H, $C^{\alpha}H$), 4.53 (q, J = 6.4 Hz, 0.7H, $C^{\alpha}H$), 5.00

(d, J = 8.2 Hz, NH, 0.6H), 5.20 (d, J = 10.4 Hz, 1H, CH=C$\underline{H}^{cis}H^{trans}$), 5.32 (d, J = 17.3 Hz, 1H, CH=C$H^{cis}\underline{H}^{trans}$), 5.93 (ddt, J = 16.5, 10.9, 5.6 Hz, 1H, C$\underline{H}$=CH_2), 6.43 (d, J = 7.4 Hz, 0.3H, NH), 6.87 (d, J = 8.3 Hz, 1H, $C^{5'}H$), 7.01 (d, J = 8.3 Hz, 1H, $C^{6'}H$), 7.19 (br m, 1H, $C^{2'}H$).

^{13}C-NMR (126 MHz, $CDCl_3$): δ (ppm) = 28.2, 28.4, 36.9, 54.4, 68.4, 69.1, 72.6, 80.5, 113.9, 117.5, 123.1, 128.7, 129.6, 131.3, 134.6, 153.6, 155.4, 175.5.

3) Synthesis of new cryptophycins

uA[acetonide]DC (**17**)

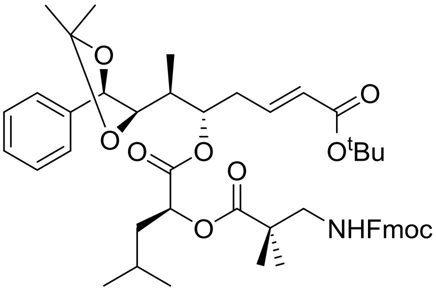

Unit A synthesized as previously described [3] (300 mg, 0.80 mmol, 1 equiv), unit CD (360 mg, 0.80 mmol, 1 equiv) and 4-DMAP (25 mg, 0.2 mmol, 0.25 equiv) were placed in a round bottomed flask under argon atmosphere. Anhydrous THF (10 mL) was added and the solution was cooled down to 0 °C. Et_3N (225 µL, 1.6 mmol, 2 equiv) and 2,4,6-trichlorobenzoyl chloride (255 µL, 1.6 mmol, 2 equiv) were added dropwise and the solution was stirred at 0 °C for 3 h. Then, a 10% citric acid solution (25 mL) was added and the solution warmed up to rt. Solution was extracted with EtOAc (3 × 50 mL), the combined organic layers were washed with sat. $NaHCO_3$ (100 mL) and brine (100 mL), dried over $MgSO_4$ and the solvent was removed under reduced pressure. The product was purified by column chromatography using PE/EtOAc (9:1 → 8:2) as eluent to obtain 520 mg (80% yield) of **17** as colourless oil.

^{1}H-NMR (600 MHz, $CDCl_3$): δ (ppm) = 0.90 (d, J = 6.5 Hz, 3H, uD-$C^{\delta}H_3$), 0.95 (d, J = 6.5 Hz, 3H, uD-$C^{\delta}H_3$), 1.09 (d, J = 7.0 Hz, 3H, uA-$C^{\varepsilon}HC\underline{H}_3$), 1.21 (s, 3H, uA-$C(CH_3)_2$), 1.23 (s, 3H, uA-$C(CH_3)_2$), 1.46 (br s, 12H, uA-$C(CH_3)_3$ and uC-$C(CH_3)_2$), 1.52 (s, 3H, uC-$C(CH_3)_2$), 1.57-1.61 (m, 1H, uD-$C^{\gamma}H$), 1.71-1.80 (m, 2H, uD-$C^{\beta}H_2$), 1.92-1.97 (m, 1H, uA-$C^{\varepsilon}H$), 2.34-2.43 (m, 2H, uA-$C^{\gamma}H_2$), 3.36 (d, J = 6.6 Hz, 2H, uC-CH_2NH), 3.83 (dd, J = 8.8, 2.5 Hz, 1H, uA-$C^{\zeta}H$), 4.23 (t, J = 7.5 Hz, 1H, C$\underline{H}CH_2$, Fmoc), 4.34 (dd, J = 10.6, 7.6 Hz, 1H, CHC$\underline{H}^AH^B$, Fmoc), 4.37 (dd, J = 10.6, 7.6 Hz, 1H, CHC$H^A\underline{H}^B$, Fmoc), 4.70 (d, J = 8.8 Hz, 1H, uA-$C^{\eta}H$),

5.01 (dd, J = 10.1, 3.5 Hz, 1H, uD-C$^{\alpha}$H), 5.05 (td, J = 6.9, 4.5 Hz, 1H, uA-C$^{\delta}$H), 5.58 (d, J = 15.6 Hz, 1H, uA-C$^{\alpha}$H), 5.98 (*t*, J = 6.6 Hz, 1H, NH), 6.58 (dt, J = 15.6, 7.3 Hz, 1H, uA-C$^{\beta}$H), 7.29-7.40 (m, 9H, uA-C^{ar}H, C^{ar}H, Fmoc), 7.63 (d, J = 7.5 Hz, 1H, C^{ar}H, Fmoc), 7.65 (d, J = 7.5 Hz, 1H, C^{ar}H, Fmoc), 7.76 (d, J = 7.5 Hz, 2H, C^{ar}H, Fmoc).

^{13}C-NMR (151 MHz, $CDCl_3$): δ (ppm) = 9.8, 21.5, 22.5, 23.1, 23.3, 25.0, 27.2, 27.3, 28.2, 33.7, 35.6, 39.6, 44.1, 47.4, 49.4, 66.9, 71.0, 75.7, 80.3, 80.4, 82.0, 109.1, 120.0, 125.4, 125.4, 126.4, 126.8, 127.1, 127.2, 127.7, 128.7, 128.9, 137.6, 141.3, 141.4, 141.4, 144.2, 144.2, 157.1, 165.4, 170.9, 176.1.

Seco-uA[acetonide]-DCB[$OCH_2CH_2OCH_2CH_2OCH_2CH_2N_3$] (**18**)

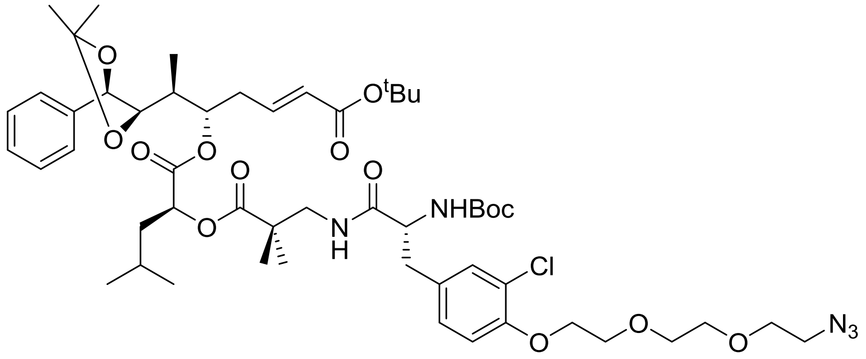

17 (290 mg, 0.35 mmol, 1 equiv) was dissolved in anhydrous DMF (2 mL) under argon atmosphere. Then, piperidine (175 µL, 1.75 mmol, 5 equiv) was added and the solution stirred at rt for 2 h. After this time the solvents were removed under reduced pressure. **13** (220 mg, 0.45 mmol, 1.3 equiv) was dissolved in anhydrous CH_2Cl_2 (1 mL) under argon atmosphere and the solution was cooled down to 0 °C. Then, Et_3N (160 µL, 1.12 mmol, 3.2 equiv) and deprotected unit ADC in anhydrous CH_2Cl_2 (1 mL) were added dropwise. Then, HOAt (78 mg, 0.57 mmol, 1.6 equiv) was added as a solid. After complete dissolution, EDC·HCl (109 mg, 0.57 mmol, 1.6 equiv), was added and the mixture stirred overnight while gradually warmed to rt. Then, H_2O (45 ml) and EtOAc (45 mL) were added, the layers were separated and the organic layer was washed with 5% $KHSO_4$ (45 mL) and saturated $NaHCO_3$ (45 mL), it was dried over $MgSO_4$ and the solvent was removed under reduced pressure. The product was purified by column chromatography using PE/EtOAc (3:2) as eluent to obtain 190 mg (51% yield) of **18** as white solid.

^{1}H-NMR (600 MHz, $CDCl_3$): δ (ppm) = 0.87 (d, J = 6.4 Hz, 3H, uD-C$^{\delta}$H$_3$), 0.90 (d, J = 6.4 Hz, 3H, uD-C$^{\delta}$H$_3$), 1.12 (d, J = 6.9 Hz, 3H, uA-C$^{\varepsilon}$HC$\underline{H}_3$), 1.15 (s, 3H, uC-C(CH_3)$_2$), 1.16 (s,

3H, uC-C(CH_3)$_2$), 1.37 (s, 9H, uB-C(CH_3)$_3$), 1.42 (s, 9H, uA-C(CH_3)$_3$), 1.45 (s, 3H, uA-C(CH_3)$_2$), 1.51 (s, 3H, uA-C(CH_3)$_2$), 1.52-1.54 (m, 1H, uD-$C^{\gamma}H$), 1.65-1.69 (m, 2H, uD-$C^{\beta}H_2$), 1.92-1.97 (m, 1H, uA-$C^{\varepsilon}H$), 2.35-2.42 (m, 2H, uA-$C^{\gamma}H_2$), 2.81 (dd, *J* = 14.0, 7.3 Hz, 1H, uB-$C^{\beta}\underline{H}^{A}H^{B}$), 3.07 (dd, *J* = 14.0, 5.8 Hz, 1H, uB-$C^{\beta}H^{A}\underline{H}^{B}$), 3.27 (dd, *J* = 13.2, 5.0 Hz, 1H, uC-$C\underline{H}^{A}H^{B}$NH), 3.38 (t, *J* = 5.1 Hz, 2H, uB-CH_2N_3), 3.50 (dd, *J* = 13.2, 7.8 Hz, 1H, uC-$CH^{A}\underline{H}^{B}$NH), 3.67-3.69 (m, 4H, uB-CH_2), 3.77-3.79 (m, 2H, uB-CH_2), 3.87-3.89 (m, 3H, uB-$C^{ar}OCH_2C\underline{H}_2$, uA-$C^{\zeta}H$), 4.14 (m, 2H, uB-$C^{ar}OC\underline{H}_2$), 4.28 (m, 1H, uB-$C^{\alpha}H$), 4.69 (d, *J* = 8.7 Hz, 1H, uA-$C^{\eta}H$), 4.91 (dd, *J* = 9.6, 3.7 Hz, 1H, uD-$C^{\alpha}H$), 5.03 (m, 1H, uA-$C^{\delta}H$), 5.19 (d, *J* = 8.1 Hz, 1H, uB-NH), 5.60 (d, *J* = 15.6 Hz, 1H, uA-$C^{\alpha}H$), 6.52 (dt, *J* = 15.4, 7.6 Hz, 1H, uA-$C^{\beta}H$), 6.84 (d, *J* = 8.3 Hz, 1H, uB-$C^{5'}H$), 7.01 (dd, *J* = 8.3, 2.1 Hz, 1H, uB-$C^{6'}H$), 7.18 (s, 1H, uB-$C^{2'}H$), 7.28-7.40 (m, 5H, uA-$C^{ar}H$).

^{13}C-NMR (151 MHz, $CDCl_3$): δ (ppm) = 9.4, 21.6, 22.5, 23.1, 23.3, 25.0, 27.1, 27.2, 28.2, 28.4, 33.6, 35.8, 38.0, 39.6, 43.8, 47.3, 50.8, 55.9, 69.1, 69.7, 70.2, 70.9, 71.1, 71.3, 75.9, 79.8, 80.4, 80.5, 82.0, 109.1, 113.8, 122.9, 126.5, 127.2, 128.7, 128.7, 128.8, 130.6, 131.4, 137.5, 141.6, 153.3, 155.2, 165.2, 171.3, 171.4, 176.0.

uA[diol]-uB[$OCH_2CH_2OCH_2CH_2OCH_2CH_2N_3$]-Cryptophycin-52 (**20**)

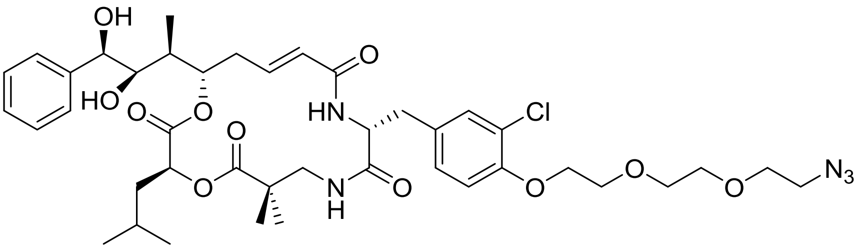

18 (165 mg, 0.16 mmol, 1 equiv) was dissolved in CH_2Cl_2 (1.75 mL), H_2O (0.2 mL) and TFA (1.70 mL) and the solution was stirred for 2 h at rt. The solvents were removed under reduced pressure and the product dried in HV overnight over KOH. The product was dissolved in DMF (10 mL) and transferred into a syringe. HATU (90 mg, 0.24 mmol, 1.5 equiv) and HOAt (33 mg, 0.24 mmol, 1.5 equiv) were dissolved in DMF (10 mL) and transferred into a second syringe. These two solutions were added to a stirred solution of DIPEA (84 μL, 0.48 mmol, 3equiv) in DMF (27.5 mL) at a rate of 0.01 mL/h using a dual channel syringe pump. Once the addition was complete, the mixture was stirred for another 2 h. Then, the solvent was removed, the crude dissolved with EtOAc (50 mL) and washed with saturated $NaHCO_3$ (50 mL) and brine (50 mL). The organic layer was dried over $MgSO_4$ and the solvent removed under reduced pressure. The product was purified by column chromatography using PE/EtOAc (1:4) as eluent to obtain 28 mg (21% yield) of **20** as white solid.

^{1}H-NMR (600 MHz, CDCl$_3$): δ (ppm) = 0.86 (d, *J* = 6.1 Hz, 3H, uD-C$^{\delta}$H$_3$), 0.92 (d, *J* = 6.1 Hz, 3H, uD-C$^{\delta}$H$_3$), 0.98 (s, 3H, uC-C(CH$_3$)$_2$), 1.00 (d, *J* = 7.0 Hz, 3H, uA-C$^{\varepsilon}$HC$\underline{H}_3$), 1.13 (s, 3H, uC-C(CH$_3$)$_2$), 1.52-1.66 (m, 4H, uD-C$^{\gamma}$H, uD-C$^{\beta}$H$_2$, uA-C$^{\varepsilon}$H), 2.15-2.20 (m, 1H, uA-C$^{\gamma}\underline{H}^{A}$H^{B}), 2.66-2.70 (m, 1H, uA-C$^{\gamma}$H$^{A}\underline{H}^{B}$), 3.01-3.05 (m, 2H, uB-C$^{\beta}$H$_2$), 3.26 (dd, *J* = 13.8, 6.1 Hz, 1H, uC-C$\underline{H}^{A}$H^{B}NH), 3.30 (dd, *J* = 13.8, 6.5 Hz, 1H, uC-CH$^{A}\underline{H}^{B}$NH), 3.38 (t, *J* = 5.1 Hz, 2H, uB-CH$_2$N$_3$), 3.67 (m, 4H, uB-CH$_2$), 3.74 (d, *J* = 8.6 Hz, 1H, uA-C$^{\zeta}$H), 3.76-3.78 (m, 2H, uB-CH$_2$), 3.89 (t, *J* = 4.9 Hz, 2H, uB-C^{ar}OCH$_2$C$\underline{H}_2$), 4.15 (t, *J* = 4.9 Hz, 2H, uB-C^{ar}OC$\underline{H}_2$), 4.32 (br, 2H, 2xOH), 4.56 (d, *J* = 8.6 Hz, 1H, uA-C$^{\eta}$H), 4.83 (q, *J* = 6.6 Hz, 1H, uB-C$^{\alpha}$H), 4.94 (td, *J* = 8.0, 3.0 Hz, 1H, uA-C$^{\delta}$H), 5.11 (dd, *J* = 10.4, 3.4 Hz, 1H, uD-C$^{\alpha}$H), 5.68 (d, *J* = 15.8 Hz, 1H, uA-C$^{\alpha}$H), 6.04 (d, *J* = 9.1 Hz, 1H, uB-NH), 6.29 (dt, *J* = 15.4, 7.2 Hz, 1H, uA-C$^{\beta}$H), 6.86 (d, *J* = 8.3 Hz, 1H, uB-C$^{5'}$H), 6.98 (t, *J* = 6.2 Hz, uC-NH), 7.01 (dd, *J* = 8.3, 2.2 Hz, 1H, uB-C$^{6'}$H), 7.18 (d, *J* = 2.2 Hz, 1H, uB-C$^{2'}$H), 7.30-7.36 (m, 5H, uA-C^{ar}H).

^{13}C-NMR (151 MHz, CDCl$_3$): δ (ppm) = 9.3, 21.3, 22.8, 23.2, 23.5, 24.9, 34.7, 35.9, 36.8, 39.1, 42.9, 46.6, 50.8, 54.0, 69.1, 69.6, 70.1, 70.8, 71.2, 74.7, 75.9, 76.8 (overlapped with solvent signal), 114.2, 123.3, 126.3, 127.0, 128.5, 128.7, 129.0, 129.9, 131.2, 139.6, 140.5, 153.6, 165.9, 171.0, 171.0, 176.3.

HPLC-MS: T$_R$ = 9.98 min, >99% purity (λ =220 nm), *m/z* = 830.38 (830.37 [M+H]$^+$)

uB[OCH$_2$CH$_2$OCH$_2$CH$_2$OCH$_2$CH$_2$N$_3$]-Cryptophycin-52 (**22**)

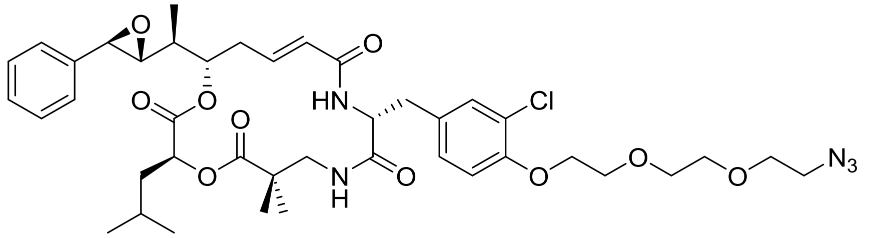

The diol of **20** (20 mg, 24.1 μmol, 1 equiv) was transformed to the corresponding epoxide using a procedure previously described in the literature [4]. Final purification by column chromatography with PE/EtOAc (1:3) as eluent and subsequent lyophilization afforded 7 mg (36% yield over 3 steps) of **22** as a white solid.

^{1}H-NMR (600 MHz, CDCl$_3$): δ (ppm) = 0.77 (d, *J* = 7.2 Hz, 3H, uD-C$^{\delta}$H$_3$), 0.78 (d, *J* = 7.2 Hz, 3H, uD-C$^{\delta}$H$_3$), 1.18 (d, *J* = 6.9 Hz, 3H, uA-C$^{\varepsilon}$HC$\underline{H}_3$), 1.20 (s, 3H, uC-C(CH$_3$)$_2$, 1.23 (s, 3H, uC-C(CH$_3$)$_2$), 1.33-1.37 (m, 2H, uD-C$^{\beta}$H$_2$), 1.50-1.52 (m, 1H, uD-C$^{\gamma}$H), 1.89-1.92 (m, 1H, uA-C$^{\varepsilon}$H), 2.43 (m, 1H, uA-C$^{\gamma}\underline{H}^{A}$H^{B}), 2.75-2.78 (m, 1H, uA-C$^{\gamma}$H$^{A}\underline{H}^{B}$), 2.81 (dd, *J* = 7.1,

1.9 Hz, 1H, uA-$C^{\zeta}H$), 3.10 (d, *J* = 6.1 Hz, 2H, uB-$C^{\beta}H_2$), 3.27 (dd, *J* = 13.3, 5.0 Hz, 1H, uC-$C\underline{H^A}H^BNH$), 3.37-3.40 (m, 2H, uC-$CH^A\underline{H^B}NH$, uB-CH_2N_3), 3.67-3.69 (m, 5H, uA-$C^{\eta}H$, uB-CH_2), 3.77-3.79 (m, 2H, uB-CH_2), 3.90 (t, *J* = 4.9 Hz, 2H, uB-$C^{ar}OCH_2C\underline{H_2}$), 4.17 (t, *J* = 4.9 Hz, 2H, uB-$C^{Ar}OC\underline{H_2}$), 4.85-4.87 (m, 1H, uB-$C^{\alpha}H$), 4.89-4.93 (m, 1H, uA-$C^{\delta}H$), 5.04 (dd, *J* = 10.7, 3.7 Hz, 1H, uD-$C^{\alpha}H$), 5.73 (d, *J* = 8.7 Hz, uB-NH), 5.78 (d, *J* = 15.5 Hz, uA-$C^{\alpha}H$), 6.46 (dt, *J* = 15.5, 7.5 Hz, uA-$C^{\beta}H$), 6.88 (d, *J* = 8.4 Hz, 1H, uB-$C^{5'}H$), 6.97 (t, *J* = 7.0 Hz, uC-NH), 7.05 (dd, *J* = 8.4, 2.2 Hz, 1H, uB-$C^{6'}H$), 7.21 (d, *J* = 2.2 Hz, 1H, uB-$C^{2'}H$), 7.24-7.25 (m, 2H, uA-$C^{ar}H$), 7.30-7.36 (m, 3H, uA-$C^{ar}H$).

^{13}C-NMR (151 MHz, $CDCl_3$): δ (ppm) = 13.5, 21.0, 23.0, 23.1, 23.7, 24.7, 34.3, 35.9, 39.1, 39.3, 43.0, 46.5, 50.9, 54.2, 59.6, 64.3, 69.2, 69.7, 70.2, 70.8, 70.9, 71.3, 76.8 (overlapped with solvent signal), 114.2, 123.3, 125.8, 127.0, 128.5, 128.8, 129.8, 131.2, 136.8, 138.4, 153.7, 165.5, 170.6, 170.7, 176.6.

HPLC-MS: T_R = 11.25 min, >99% purity (λ =220 nm), *m/z* = 812.37 (812.36 $[M+H]^+$)

HRMS (ESI-MS): *m/z* calculated for $C_{41}H_{55}ClN_5O_{10}$ $[M+H]^+$ 812.3632; found 812.3626

Seco-uA[acetonide]-DCB[$OCH_2CH_2OCH_2CHCH_2$] (**19**)

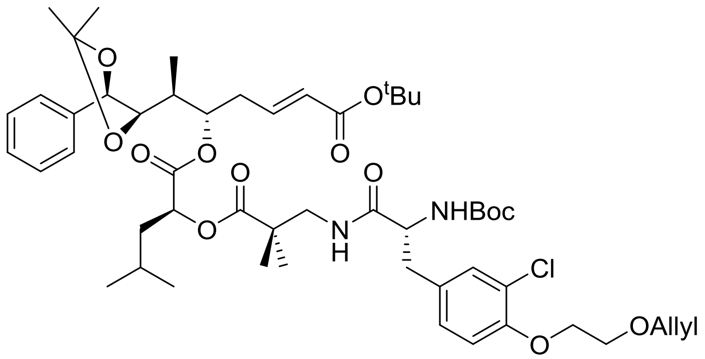

17 (500 mg, 0.62 mmol, 1 equiv) was dissolved in anhydrous DMF (4 mL) under argon atmosphere. Then, piperidine (305 µL, 3.1 mmol, 5 equiv) was added and the solution stirred at rt for 2 h. After this time the solvents were removed under reduced pressure. **14** (320 mg, 0.81 mmol, 1.3 equiv) was dissolved in anhydrous CH_2Cl_2 (2 mL) under argon atmosphere and the solution was cooled down to 0 °C. Then, Et_3N (275 µL, 1.97 mmol, 3.2 equiv) and deprotected unit ADC in anhydrous CH_2Cl_2 (2 mL) were added dropwise. Then, HOAt (135 mg, 0.99 mmol, 1.6 equiv) was added as a solid. After complete dissolution, EDC·HCl (190 mg, 0.99 mmol, 1.6 equiv), was added and the mixture stirred overnight while gradually warmed to rt. Then, H_2O (75 ml) and EtOAc (75 mL) were added, the layers were separated and the organic

layer was washed with 5% $KHSO_4$ (75 mL) and saturated $NaHCO_3$ (75 mL), it was dried over $MgSO_4$ and the solvent was removed under reduced pressure. The product was purified by column chromatography using PE/EtOAc (7:3) as eluent to obtain 350 mg (59% yield) of **19** as white solid.

^{1}H-NMR (600 MHz, $CDCl_3$): δ (ppm) = 0.84 (d, *J* = 6.3 Hz, 3H, uD-$C^{\delta}H_3$), 0.91 (d, *J* = 6.3 Hz, 3H, uD-$C^{\delta}H_3$), 1.08 (s, 3H, uC-$C(CH_3)_2$), 1.11 (d, *J* = 7.0 Hz, 3H, uA-$C^{\varepsilon}HC\underline{H_3}$), 1.17 (s, 3H, uC-$C(CH_3)_2$), 1.34 (s, 9H, uB-$C(CH_3)_3$), 1.43 (s, 3H, uA-$C(CH_3)_2$), 1.47 (s, 9H, uA-$C(CH_3)_3$), 1.48 (s, 3H, uA-$C(CH_3)_2$), 1.66-1.74, m, 3H, uD-$C^{\gamma}H$, uD-$C^{\beta}H_2$), 1.85-1.90 (m, 1H, uA-$C^{\varepsilon}H$), 2.27-2.33 (m, 1H, uA-$C^{\gamma}H_2$), 2.50-2.54 (m, 1H, uA-$C^{\gamma}H_2$), 2.83 (dd, *J* = 13.6, 9.9 Hz, 1H, uB-$C^{\beta}\underline{H^A}H^B$), 3.22-3.28 (m, 2H, uB-$C^{\beta}H^A\underline{H^B}$, uC-$C\underline{H^A}H^BNH$), 3.61 (dd, *J* = 13.4, 8.3 Hz, 1H, uC-$CH^A\underline{H^B}NH$), 3.81-3.83 (m, 3H, $C^{ar}OCH_2C\underline{H_2}$, uA-$C^{\zeta}H$), 4.12 (dt, *J* = 5.7, 1.4 Hz, 2H, $C^{ar}O(CH_2)_2OC\underline{H_2}$), 4.14 (t, *J* = 5.0 Hz, 2H, $C^{ar}OC\underline{H_2}$), 4.41 (q, *J* = 9.1 Hz, 1H, uB-$C^{\alpha}H$), 4.70 (d, *J* = 8.9 Hz, 1H, uA-$C^{\eta}H$), 4.89 (dd, *J* = 10.2, 3.0 Hz, 1H, uD-$C^{\alpha}H$), 5.08 (t, *J* = 8.5 Hz, 1H, uA-$C^{\delta}H$), 5.19 (dq, *J* = 10.3, 1.4 Hz, 1H, $CH=CH^{trans}\underline{H^{cis}}$), 5.31 (dq, *J* = 17.3, 1.6 Hz, 1H, $CH=C\underline{H^{trans}}H^{cis}$), 5.68 (d, *J* = 15.5 Hz, 1H, uA-$C^{\alpha}H$), 5.79 (d, *J* = 8.7 Hz, 1H, uB-NH), 5.92 (ddt, *J* = 17.3, 10.8, 5.6 Hz, 1H, $C\underline{H}=CH_2$), 6.74 (ddd, *J* = 15.2, 8.9, 5.3 Hz, 1H, uA-$C^{\beta}H$), 6.85 (d, *J* = 8.3 Hz, 1H, uB-$C^{5'}H$), 7.09 (dd, *J* = 8.5, 2.1 Hz, 1H, uB-$C^{6'}H$), 7.13-7.15 (m, 1H, uC-NH), 7.26-7.37 (m, 6H, uB-$C^{2'}H$, uA-$C^{ar}H$).

^{13}C-NMR (151 MHz, $CDCl_3$): δ (ppm) = 9.7, 21.3, 22.0, 23.3, 24.9, 27.2, 27.3, 28.4, 28.4, 34.9, 35.7, 37.7, 39.5, 44.0, 47.6, 56.3, 60.5, 68.4, 69.2, 70.5, 72.6, 75.6, 79.6, 80.2, 80.5, 82.0, 109.1, 114.0, 117.3, 122.9, 125.9, 126.7, 128.6, 128.9, 131.4, 131.6, 134.8, 137.7, 142.1, 153.2, 155.9, 166.0, 170.9, 172.0, 175.6.

uA[diol]-uB[$OCH_2CH_2OCH_2CHCH_2$]-Cryptophycin-52 (**21**)

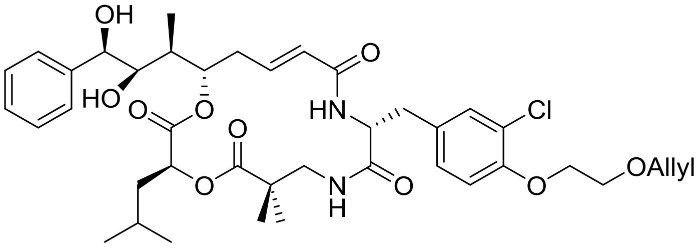

19 (310 mg, 0.32 mmol, 1 equiv) was dissolved in CH_2Cl_2 (3. 5 mL), H_2O (0.35 mL) and TFA (3.5 mL) and the solution was stirred for 2 h at rt. The solvents were removed under reduced pressure and the product dried in HV overnight over KOH. The product was dissolved in DMF (16.7 mL) and transferred

into a syringe. HATU (182 mg, 0.48 mmol, 1.5 equiv) and HOAt (65 mg, 0.48 mmol, 1.5 equiv) were dissolved in DMF (16.7 mL) and transferred into a second syringe. These two solutions were added to a stirred solution of DIPEA (168 µL, 0.96 mmol, 3 equiv) in DMF (15 mL) at a rate of 0.01 mL/h using a dual channel syringe pump. Once the addition was complete, the mixture was stirred for another 2 h. Then, the solvent was removed, the crude dissolved with EtOAc (100 mL) and washed with saturated $NaHCO_3$ (100 mL) and brine (100 mL). The organic layer was dried over $MgSO_4$ and the solvent was removed under reduced pressure. The product was purified by column chromatography using PE/EtOAc (1:4) as eluent to obtain 60 mg (25% yield) of **21** as white solid.

^{1}H-NMR (600 MHz, $CDCl_3$): δ (ppm) = 0.85 (d, *J* = 6.6 Hz, 3H, uD-$C^{\delta}H_3$), 0.91 (d, *J* = 6.6 Hz, 3H, uD-$C^{\delta}H_3$), 0.97 (d, *J* = 7.0 Hz, 3H, uA-$C^{\varepsilon}HC\underline{H}_3$), 1.14 (s, 3H, uC-$C(CH_3)_2$), 1.21 (s, 3H, uC-$C(CH_3)_2$), 1.41-1.47 (m, 2H, uA-$C^{\varepsilon}H$, uD-$C^{\beta}H_2$), 1.60-1.64 (m, 1H, uD-$C^{\gamma}H$), 1.74-1.79 (m, 1H, uD-$C^{\beta}H_2$), 2.18-2.24 (m, 1H, uA-$C^{\gamma}H_2$), 2.39-2.43 (m, 1H, uA-$C^{\gamma}H_2$), 2.90 (dd, *J* = 14.6, 9.3 Hz, 1H, uB-$C^{\beta}\underline{H^A}H^B$), 3.08 (dd, *J* = 14.6, 4.8 Hz, 1H, uB-$C^{\beta}H^A\underline{H^B}$), 3.13 (dd, *J* = 13.5, 3.9 Hz, 1H, uC-$C\underline{H^A}H^BNH$), 3.33 (dd, *J* = 13.5, 8.2 Hz, 1H, uC-$CH^A\underline{H^B}NH$), 3.50 (br, 1H, uA-OH), 3.67 (br, 1H, uA-OH), 3.75 (d, *J* = 8.3 Hz, 1H uA-$C^{\zeta}H$), 3.81 (t, *J* = 4.9 Hz, 2H, $C^{ar}OCH_2C\underline{H_2}$), 4.09-4.12 (m, 4H, $C^{ar}O(CH_2)_2OC\underline{H_2}$, $C^{ar}OC\underline{H_2}$), 4.53 (d, *J* = 8.3 Hz, 1H, uA-$C^{\eta}H$), 4.68 (td, *J* = 8.2, 4.9 Hz, 1H, uB-$C^{\alpha}H$), 4.85 (dd, *J* = 10.1, 3.6 Hz, 1H, uD-$C^{\alpha}H$), 5.03-5.06 (m, 1H, uA-$C^{\delta}H$), 5.18 (dq, *J* = 10.4, 1.5 Hz, 1H, $CH{=}CH^{trans}\underline{H^{cis}}$), 5.30 (dq, *J* = 17.4, 1.7 Hz, 1H, $CH{=}C\underline{H^{trans}}H^{cis}$), 5.72 (d, *J* = 15.2 Hz, 1H, uA-$C^{\alpha}H$), 5.91 (ddt, *J* = 17.2, 10.8, 5.5 Hz, 1H, $C\underline{H}{=}CH_2$), 6.18 (d, *J* = 7.8 Hz, 1H, uB-NH), 6.68 (ddd, *J* = 15.1, 10.8, 4.2 Hz, 1H, uA-$C^{\beta}H$), 6.82 (d, *J* = 8.4 Hz, 1H, uB-$C^{5'}H$), 7.00 (dd, *J* = 8.4, 2.1 Hz, 1H, uB-$C^{6'}H$), 7.16 (d, *J* = 2.1 Hz, 1H, uB-$C^{2'}H$), 7.24-7.32 (m, 6H, uA-$C^{ar}H$, uC-NH).

^{13}C-NMR (151 MHz, CDCl3): δ (ppm) = 9.7, 21.7, 22.9, 23.1, 23.1, 24.9, 35.3, 36.2, 38.1, 39.7, 42.8, 46.6, 54.7, 68.4, 69.1, 71.2, 72.5, 74.9, 75.8, 76.6, 114.0, 117.3, 123.1, 124.5, 127.0, 128.2, 128.3, 128.7, 130.4, 130.9, 134.7, 140.9, 142.7, 153.5, 165.7, 170.6, 170.9, 177.6.

HPLC-MS: T_R = 10.35 min, >99% purity (λ =220 nm), *m/z* = 757.35 (757.35 $[M+H]^+$)

uB[$OCH_2CH_2OCH_2CHCH_2$]-Cryptophycin-52 (**23**)

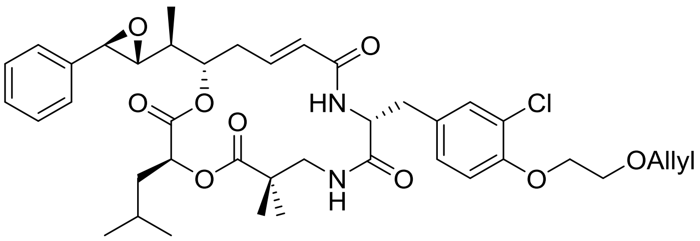

The diol of **21** (40 mg, 52.9 µmol, 1 equiv) was transformed to the corresponding epoxide using a procedure previously described in the literature [4]. Final purification by column chromatography with PE/EtOAc (1:2) as eluent and subsequent lyophilization afforded 24 mg (61% yield over 3 steps) of **23** as a white solid.

^{1}H-NMR (600 MHz, $CDCl_3$): δ (ppm) = 0.82 (d, J = 6.5 Hz, 3H, uD-$C^{\delta}H_3$), 0.84 (d, J = 6.5 Hz, 3H, uD-$C^{\delta}H_3$), 1.14 (d, J = 6.9 Hz, 3H, uA-$C^{\varepsilon}HC\underline{H}_3$), 1.15 (s, 3H, uC-$C(CH_3)_2$), 1.21 (s, 3H, uC-$C(CH_3)_2$), 1.28-1.32 (m, 1H, uD-$C^{\gamma}H$), 1.62-1.73 (m, 2H, uD-$C^{\beta}H_2$), 1.75-1.81 (m, 1H, uA-$C^{\varepsilon}H$), 2.41-2.47 (m, 1H, uA-$C^{\gamma}H_2$), 2.55-2.59 (m, 1H, uA-$C^{\gamma}H_2$), 2.92 (dd, J = 7.5, 2.0 Hz, 1H, uA-$C^{\zeta}H$), 3.02 (dd, J = 14.5, 7.7 Hz, 1H, uB-$C^{\beta}\underline{H^A}H^B$), 3.08 (m, 2H, uB-$C^{\beta}H^A\underline{H^B}$, uC-$C\underline{H^A}H^BNH$), 3.40 (dd, J = 13.6, 8.6 Hz, 1H, uC-$CH^A\underline{H^B}NH$), 3.68 (d, J = 2.0 Hz, 1H, uA-$C^{\eta}H$), 3.83 (t, J = 4.9 Hz, $C^{ar}OCH_2C\underline{H_2}$), 4.12 (dt, J = 5.6, 1.5 Hz, 2H, $C^{ar}O(CH_2)_2OC\underline{H_2}$), 4.15 (t, J = 4.9 Hz, 2H, $C^{ar}OC\underline{H_2}$), 4.72 (td, J = 7.7, 4.9 Hz, 1H, uB-$C^{\alpha}H$), 4.83 (dd, J = 10.2, 3.5 Hz, 1H, uD-$C^{\alpha}H$), 5.17-5.21 (m, 2H, uA-$C^{\delta}H$, $CH=CH^{trans}\underline{H^{cis}}$), 5.32 (dq, J = 17.4, 1.7 Hz, 1H, $CH=C\underline{H^{trans}}H^{cis}$), 5.63 (d, J = 7.8 Hz, uB-NH), 5.71 (dd, J = 15.1, 1.8 Hz, 1H, uA-$C^{\alpha}H$), 5.93 (ddt, J = 17.3, 10.8, 5.6 Hz, 1H, $C\underline{H}=CH_2$), 6.75 (ddd, J = 15.0, 10.6, 4.3 Hz, 1H, uA-$C^{\beta}H$), 6.85 (d, J = 8.4 Hz, 1H, uB-$C^{5'}H$), 7.01 (dd, J = 8.4, 2.2 Hz, 1H, uB-$C^{6'}H$), 7.18 (d, J = 2.2 Hz, 1H, uB-$C^{2'}H$), 7.21 (dd, J = 8.9, 3.5 Hz, 1H, uC-NH), 7.23-7.25 (m, 2H, uA-$C^{ar}H$), 7.31-7.37 (m, 3H, uA-$C^{ar}H$).

^{13}C-NMR (151 MHz, CDCl3): δ (ppm) = 13.7, 21.4, 22.9, 23.0, 24.7, 35.5, 37.0, 39.5, 40.8, 42.9, 46.6, 54.5, 59.2, 63.2, 68.4, 69.2, 71.3, 72.6, 76.0, 114.2, 117.4, 123.4, 124.8, 125.7, 128.3, 128.7, 128.9, 130.0, 131.0, 134.7, 136.9, 141.9, 153.7, 165.1, 170.5, 170.6, 178.1.

HPLC-MS: T_R = 11.53 min, 98% purity (λ =220 nm), m/z = 739.33 (739.34 $[M+H]^+$)

HRMS (ESI-MS): m/z calculated for $C_{40}H_{52}ClN_2O_9$ $[M+H]^+$ 739.3356; found 739.3359

uB[OCH_2CH_2OH]-Cryptophycin-52 (**24**)

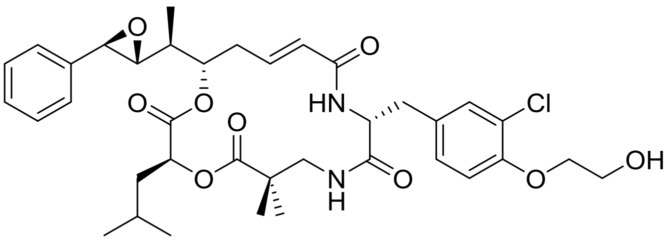

23 (5 mg, 6.8 µmol, 1 equiv) and $Pd(PPh_3)_4$ (0.8 mg, 0.68 µmol, 10%) were dissolved with anhydrous and degassed CH_2Cl_2 (1 mL). Phenylsilane (4.2 µL, 34 µmol, 5 equiv) was added and the solution was stirred at rt for 7 h. Then, the solvent was removed by bubbling air and the product was purified by column chromatography using PE/EtOAc (1:2) as eluent and subsequent lyophilization afforded 2 mg (42% yield) of **24** as white solid.

^{1}H-NMR (600 MHz, $CDCl_3$): δ (ppm) = 0.83 (d, *J* = 6.4 Hz, 3H, uD-$C^{\delta}H_3$), 0.84 (d, *J* = 6.4 Hz, 3H, uD-$C^{\delta}H_3$), 1.14-1.15 (m, 6H, uA-$C^{\varepsilon}HC\underline{H_3}$, uC-$C(CH_3)_2$), 1.22 (s, 3H, uC-$C(CH_3)_2$), 1.30-1.34 (m, 1H, uD-$C^{\gamma}H$), 1.66-1.74 (m, 2H, uD-$C^{\beta}H_2$), 1.77-1.80 (m, 1H, uA-$C^{\varepsilon}H$), 2.14-2.18 (br s, 1H, OH), 2.42-2.48 (m, 1H, uA-$C^{\gamma}H_2$), 2.56-2.59 (m, 1H, uA-$C^{\gamma}H_2$), 2.92 (d, *J* = 7.3 Hz, 1H, uA-$C^{\zeta}H$), 3.05-3.13 (m, 3H, uB-$C^{\beta}H_2$, uC-$C\underline{H^A}H^BNH$), 3.40 (dd, *J* = 13.6, 8.3 Hz, 1H, uC-$CH^A\underline{H^B}NH$), 3.68 (s, 1H, uA-$C^{\eta}H$), 3.98 (s, 2H, $C^{ar}OCH_2C\underline{H_2}$), 4.12 (t, *J* = 4.1 Hz, 2H, $C^{ar}OC\underline{H_2}$), 4.75 (q, *J* = 6.5 Hz, 1H, uB-$C^{\alpha}H$), 4.83 (dd, *J* = 10.0, 3.7 Hz, 1H, uD-$C^{\alpha}H$), 5.19-5.21 (m, 1H, uA-$C^{\delta}H$), 5.44 (d, *J* = 7.8 Hz, uB-NH), 5.72 (d, *J* = 15.2 Hz, 1H, uA-$C^{\alpha}H$), 6.76 (ddd, *J* = 15.0, 10.5, 4.4 Hz, 1H, uA-$C^{\beta}H$), 6.86 (d, *J* = 8.2 Hz, 1H, uB-$C^{5'}H$), 7.04 (d, *J* = 8.2 Hz, 1H, uB-$C^{6'}H$), 7.18-7.25 (m, 3H, uB-$C^{2'}H$, uA-$C^{ar}H$), 7.34-7.38 (m, 3H, uA-$C^{ar}H$).

HPLC-MS: T_R = 10.61 min, >99% purity (λ =220 nm), *m/z* = 699.31 (699.30 $[M+H]^+$)

HRMS (ESI-MS): *m/z* calculated for $C_{37}H_{48}ClN_2O_9$ $[M+H]^+$ 699.3043; found 699.3047

4) NMR, HPLC and mass spectra

4: ^{1}H-NMR and ^{13}C-NMR

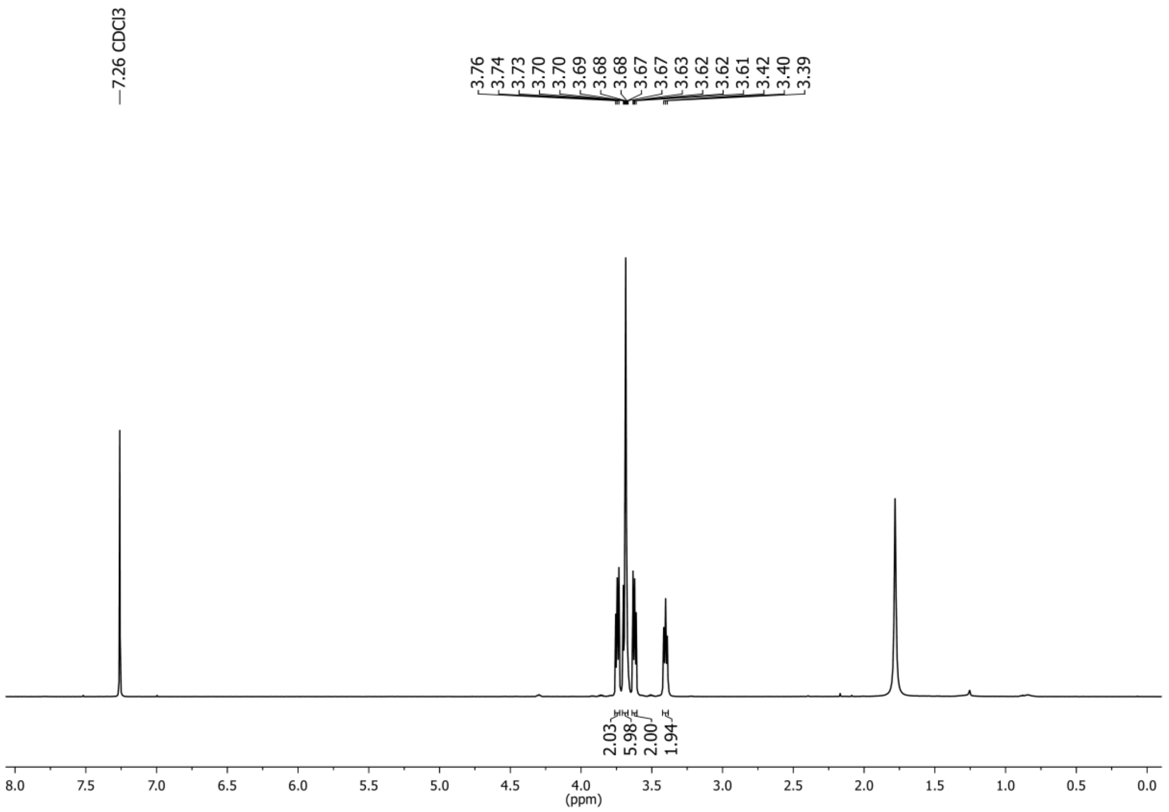

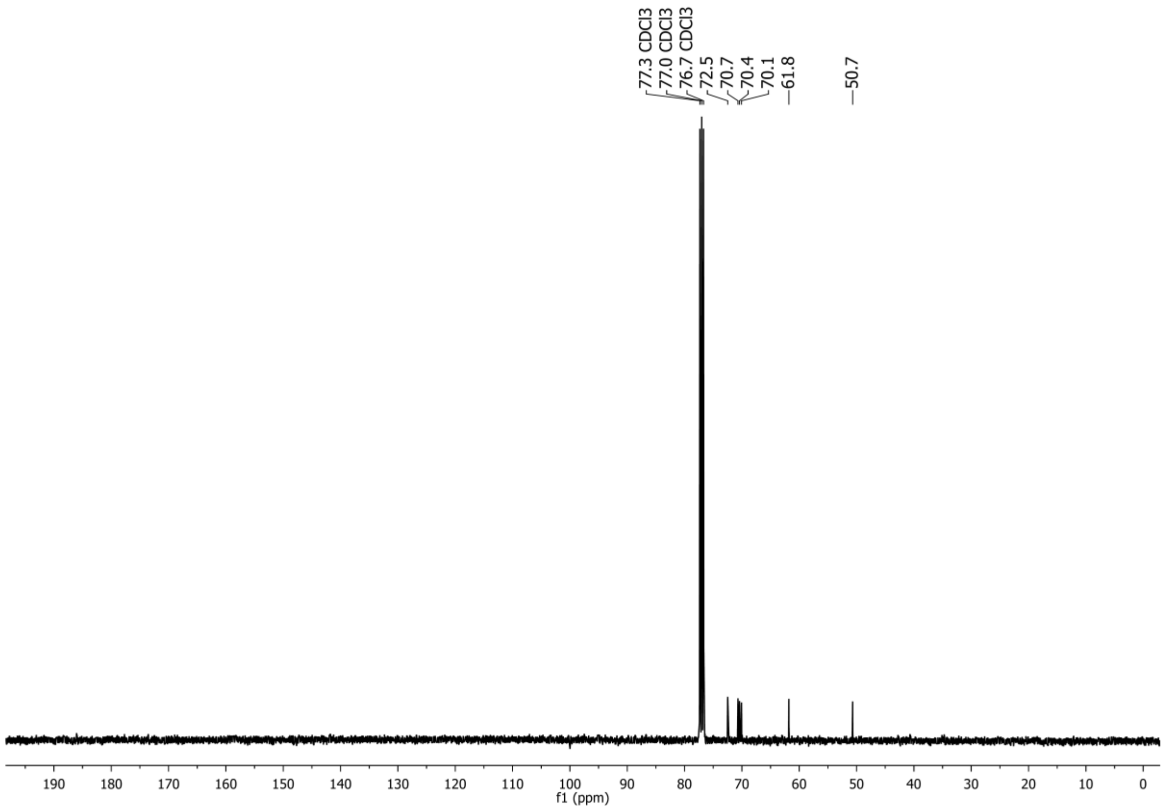

5: ^{1}H-NMR and ^{13}C-NMR

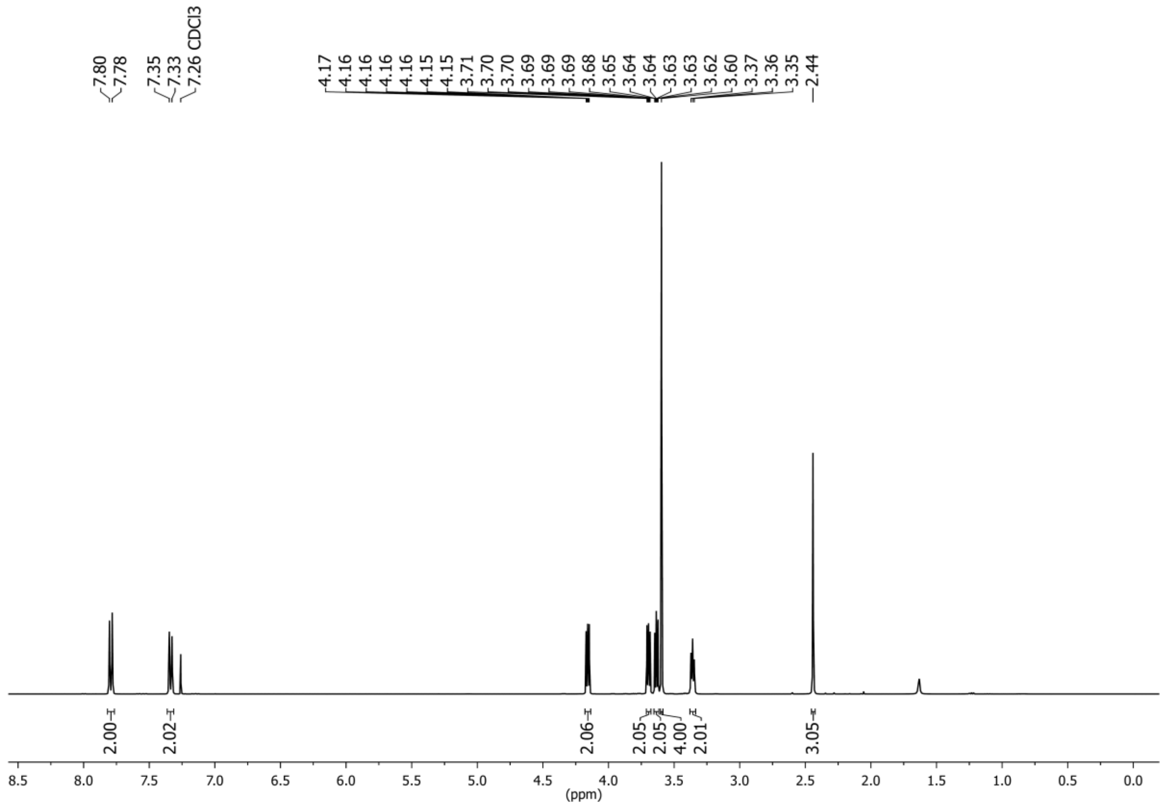

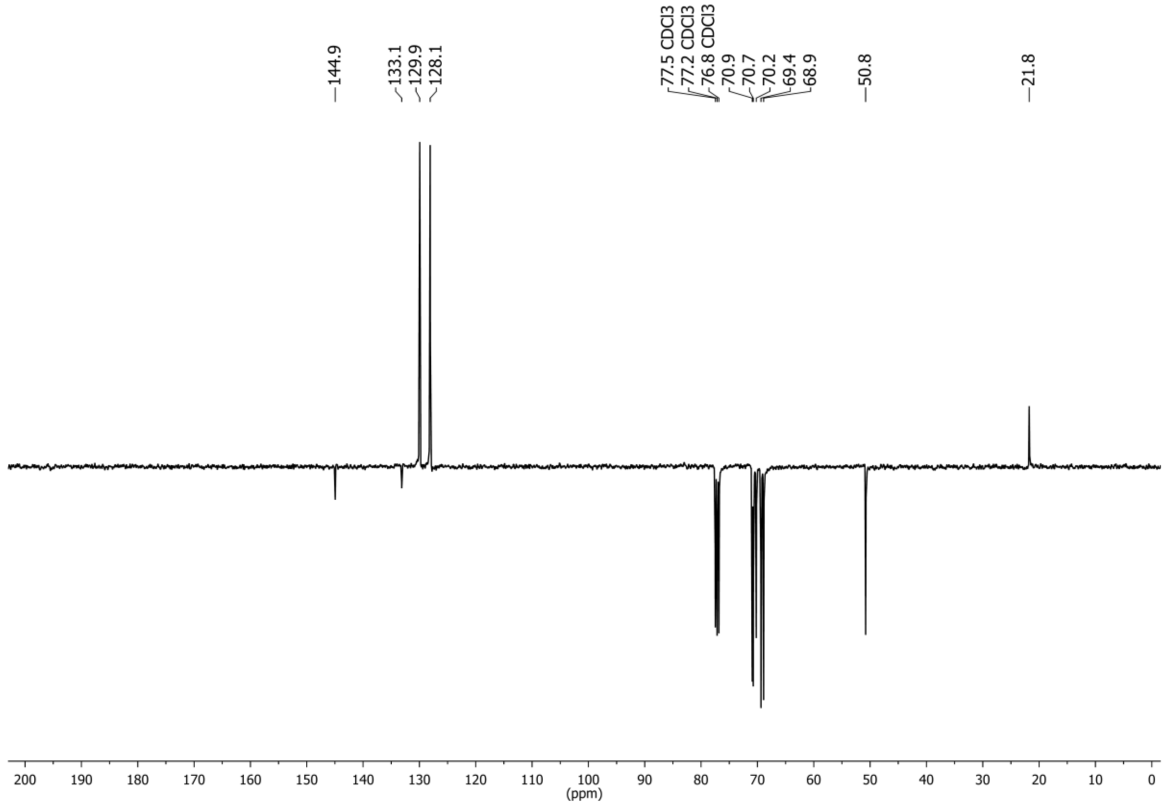

6: ^{1}H-NMR

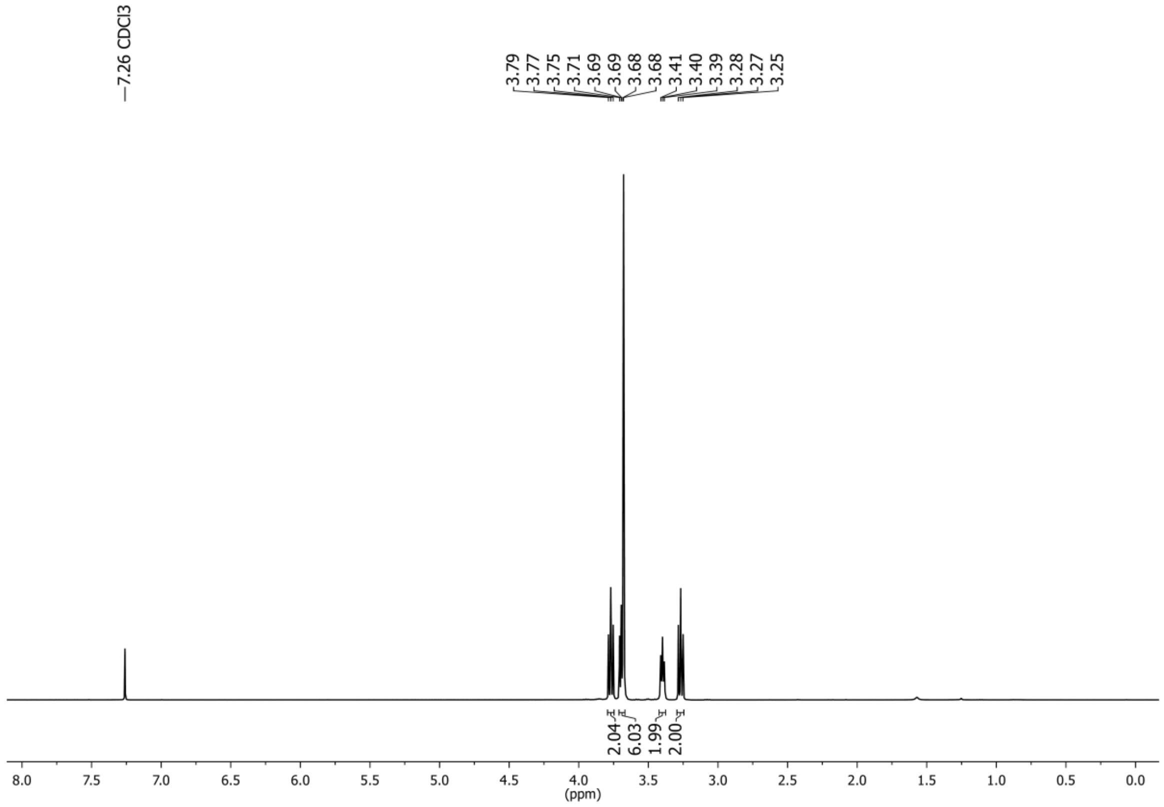

7.26 CDCl3
3.79
3.77
3.75
3.71
3.69
3.69
3.68
3.68
3.41
3.40
3.39
3.28
3.27
3.25
2.04
6.03
1.99
2.00
8.0 7.5 7.0 6.5 6.0 5.5 5.0 4.5 4.0 3.5 3.0 2.5 2.0 1.5 1.0 0.5 0.0
(ppm)

8: ^{1}H-NMR and ^{13}C-NMR

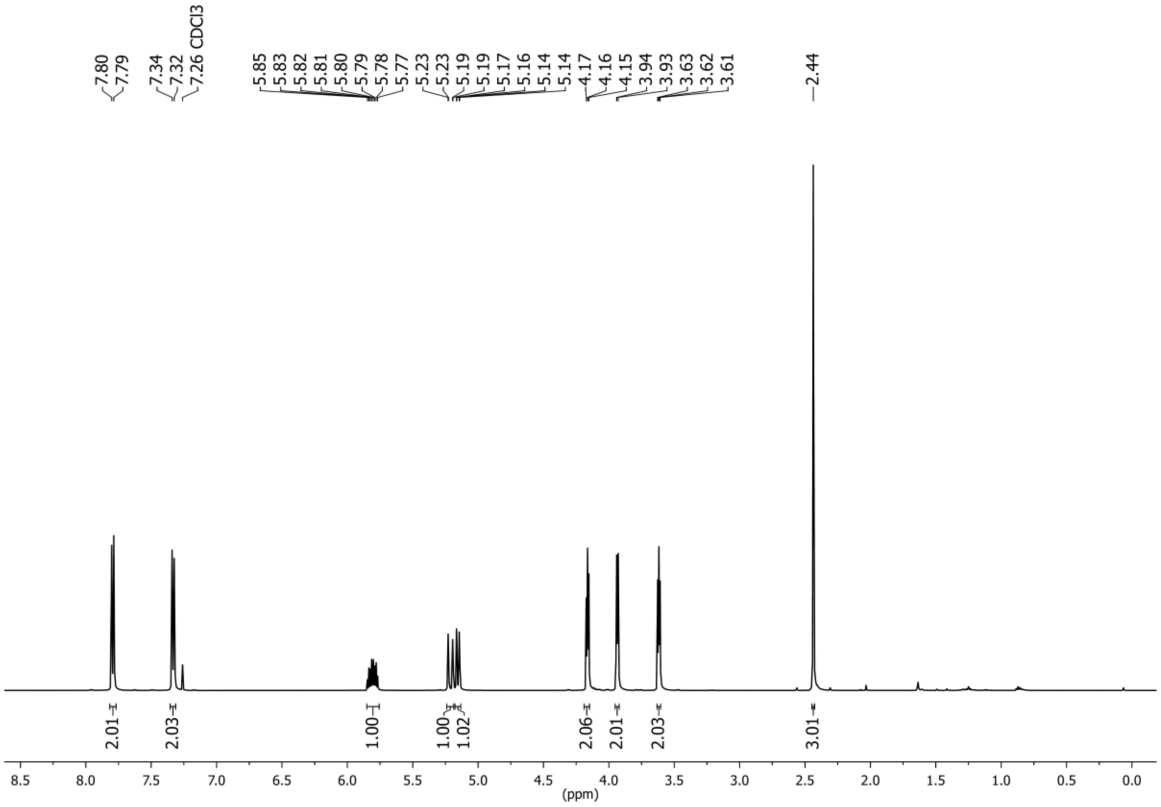

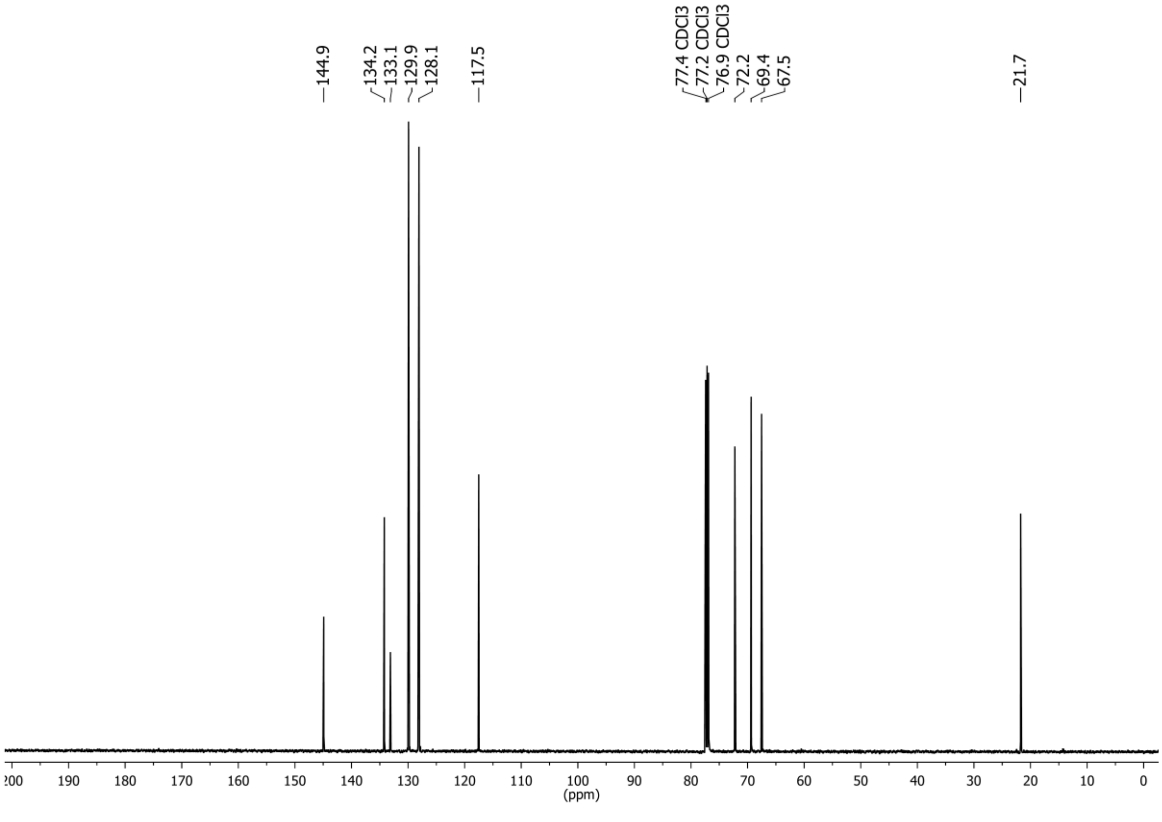

9: ^{1}H-NMR and ^{13}C-NMR

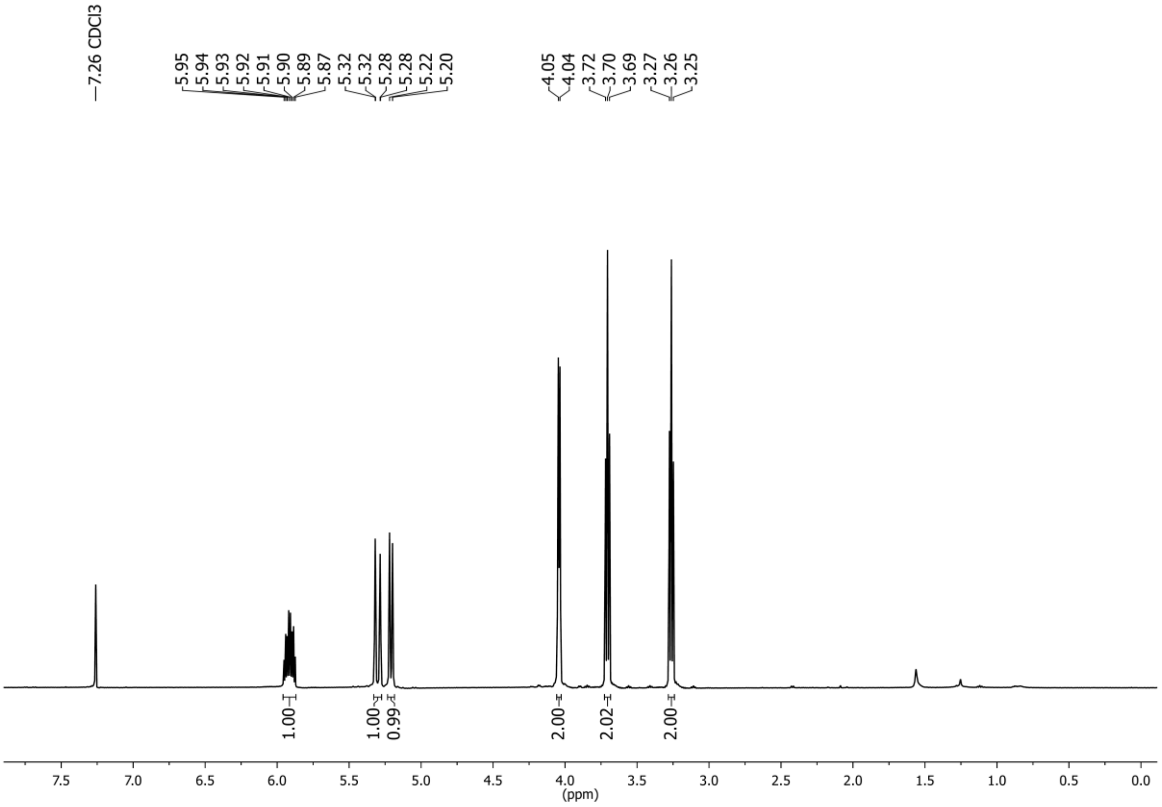

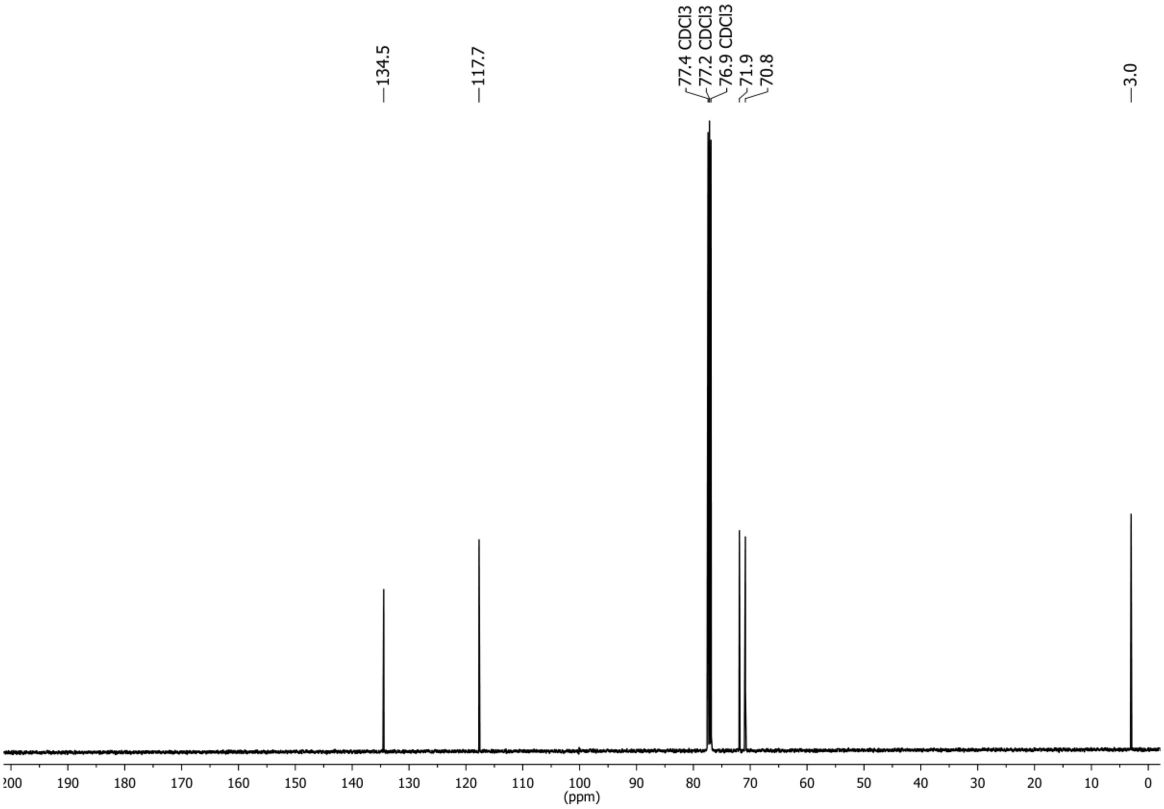

11: ^{1}H-NMR and ^{13}C-NMR

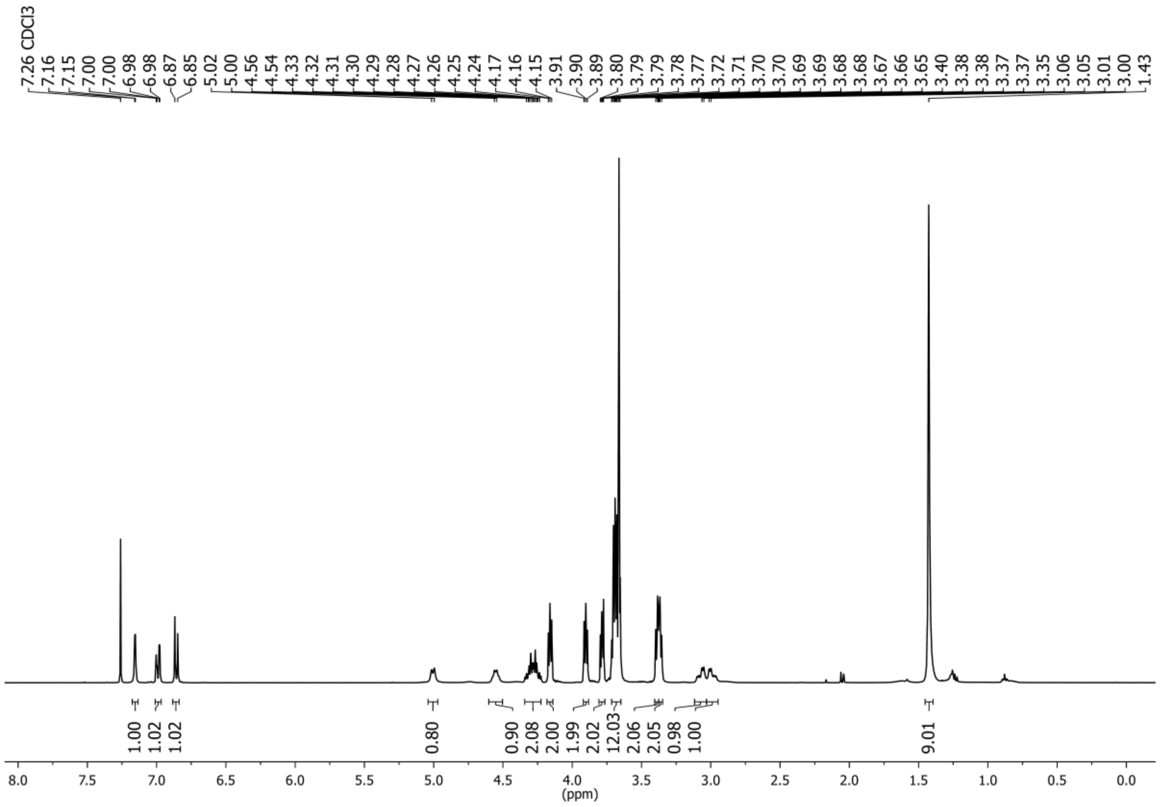

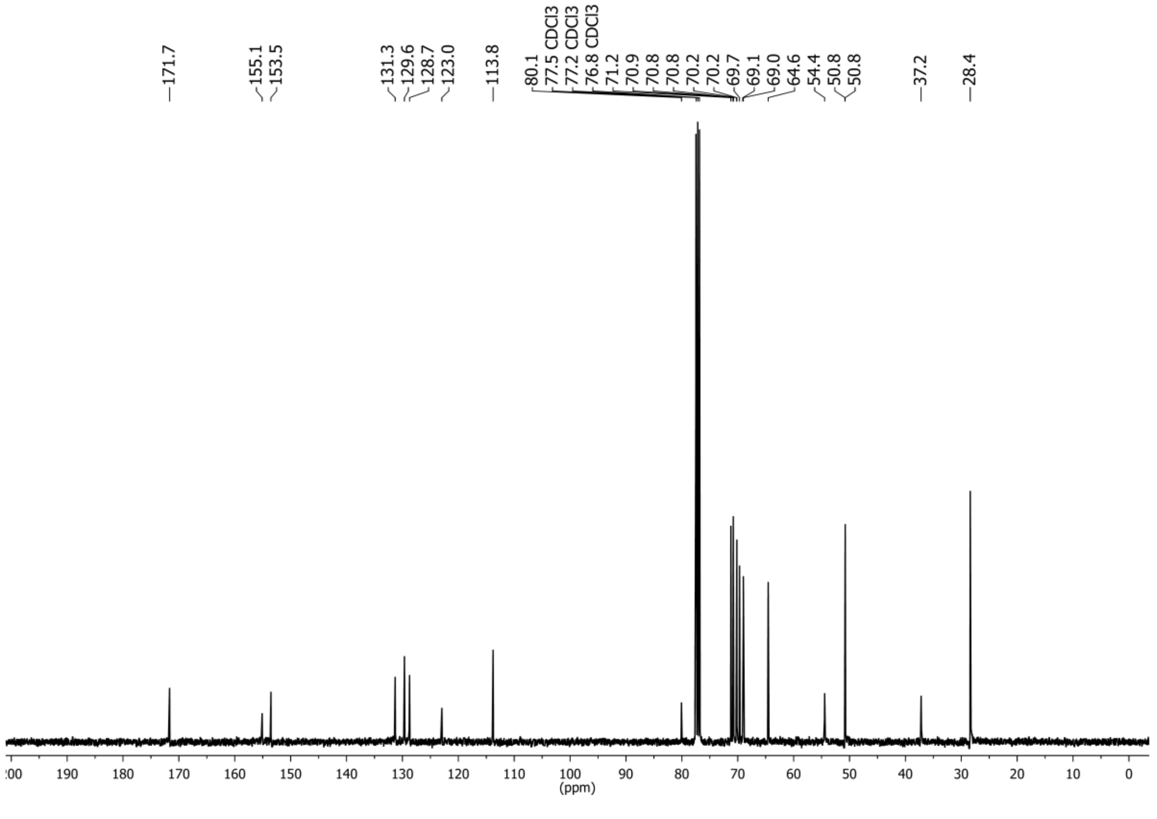

13: ^{1}H-NMR and ^{13}C-NMR

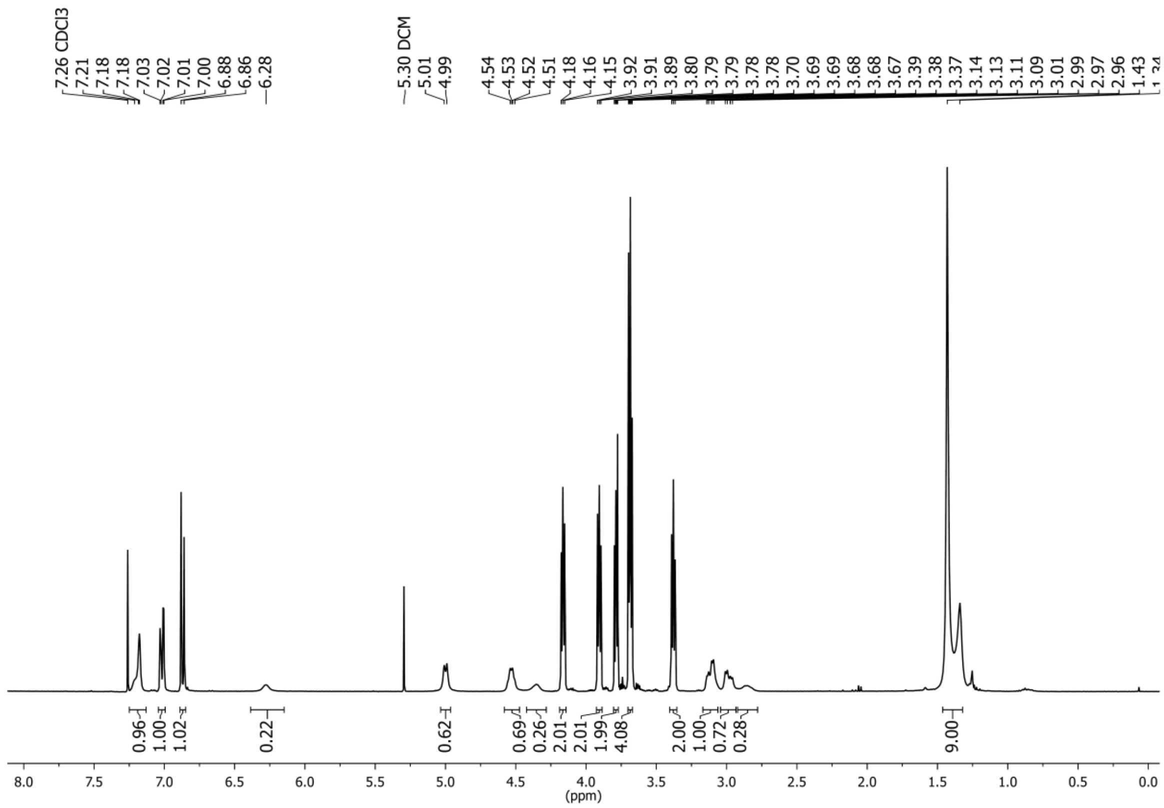

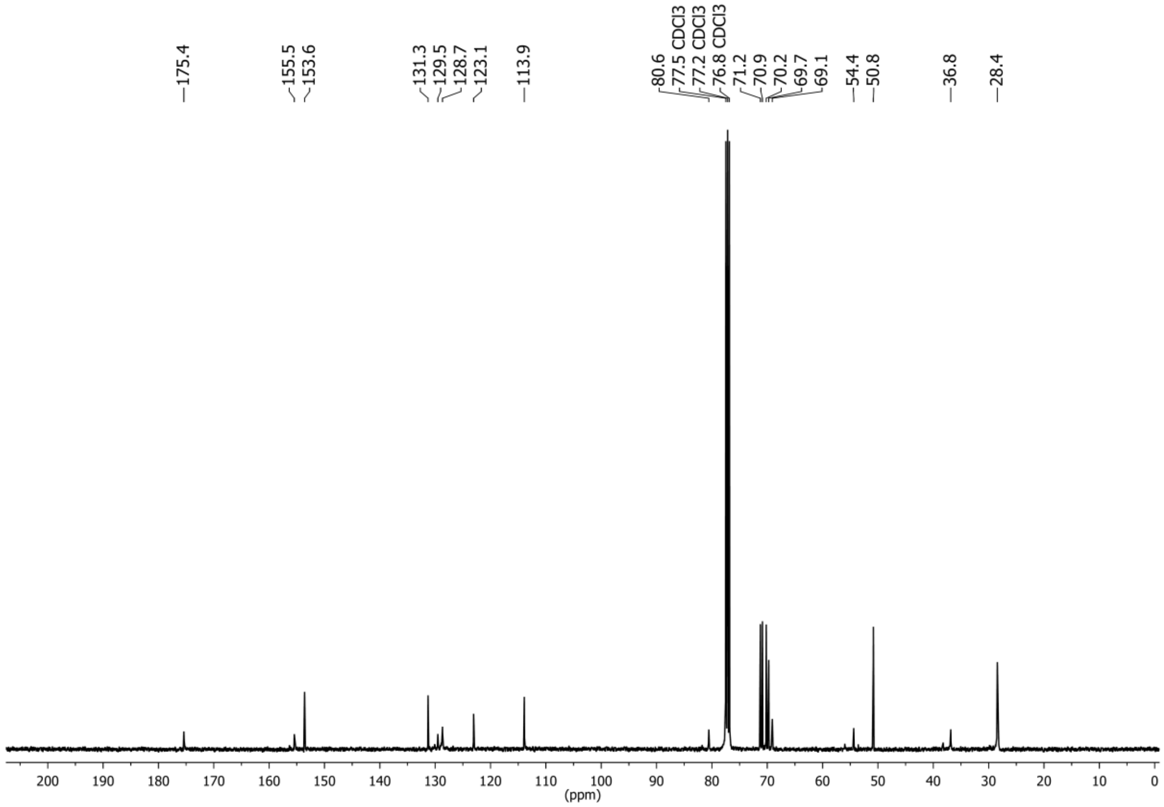

12: ^{1}H-NMR and ^{13}C-NMR

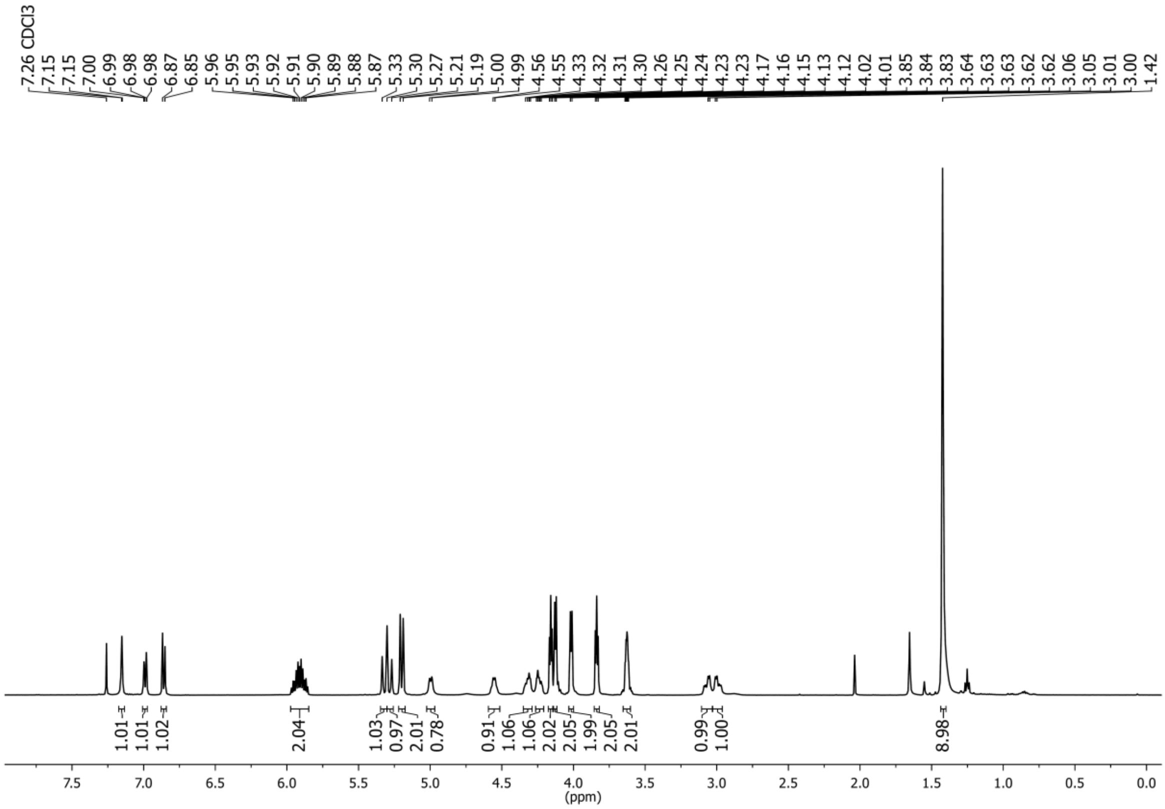

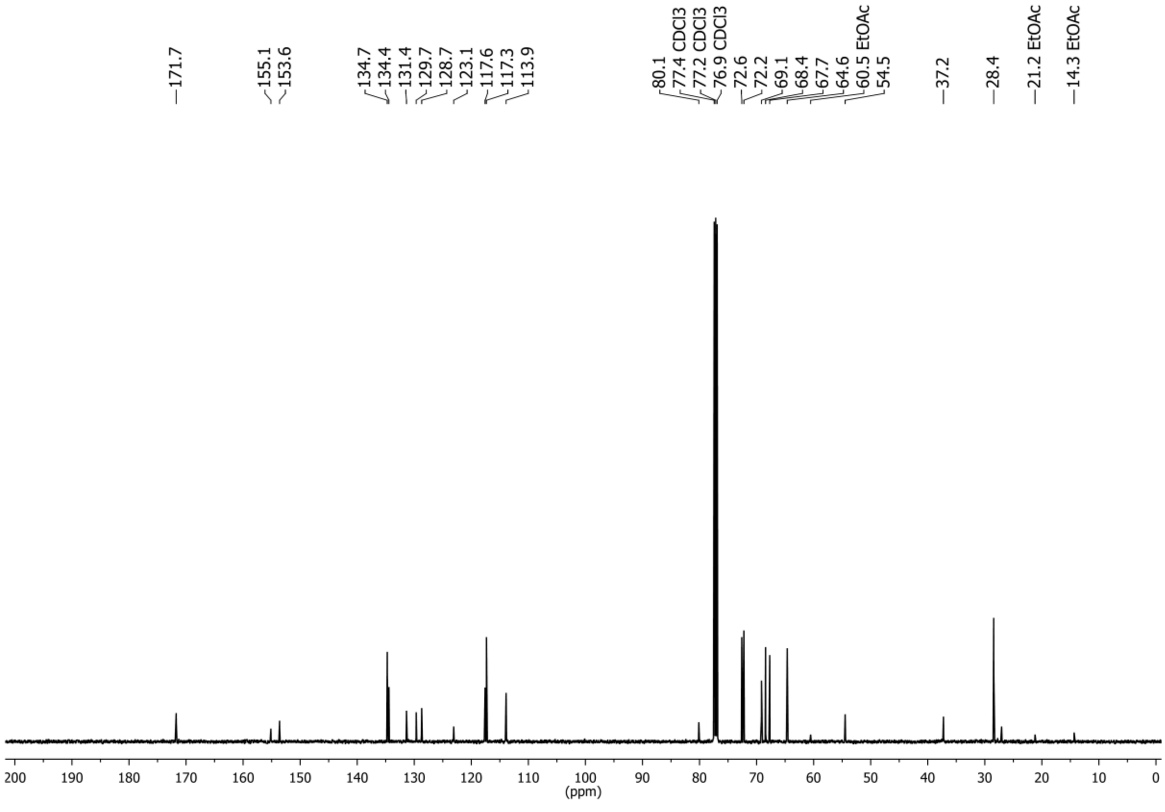

14: ^{1}H-NMR and ^{13}C-NMR

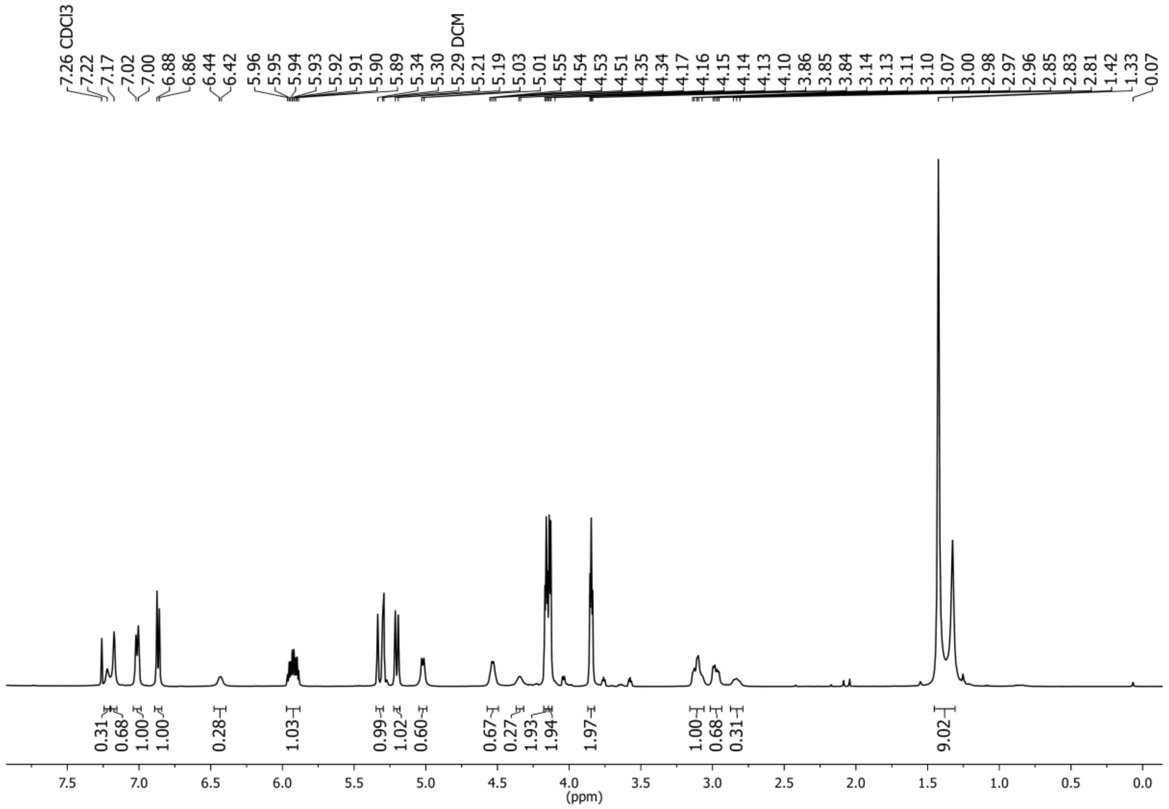

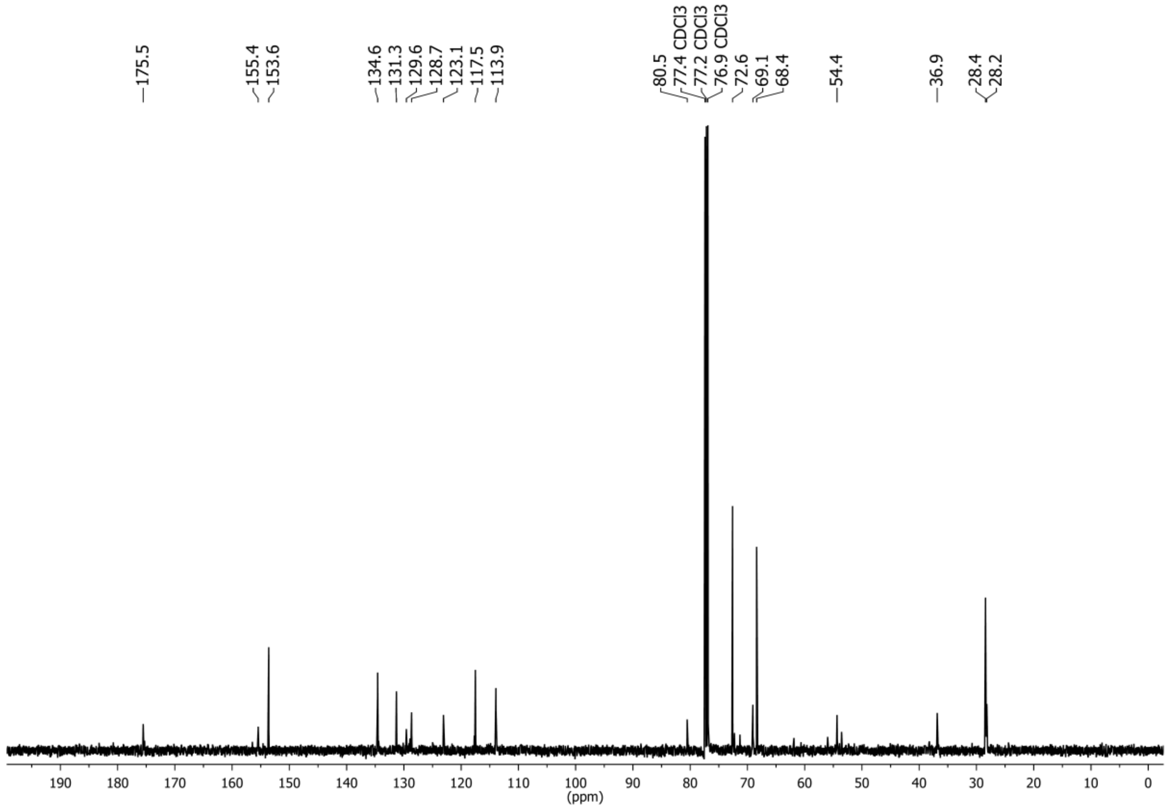

17: ^{1}H-NMR and ^{13}C-NMR

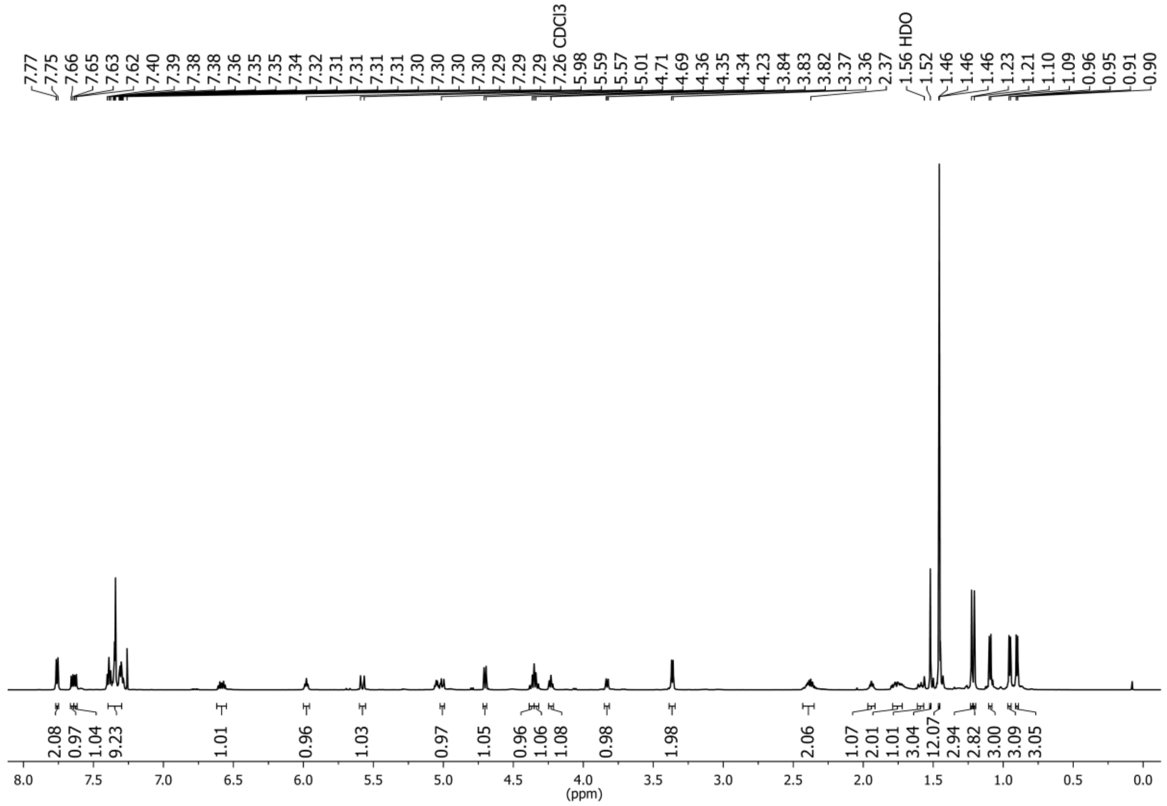

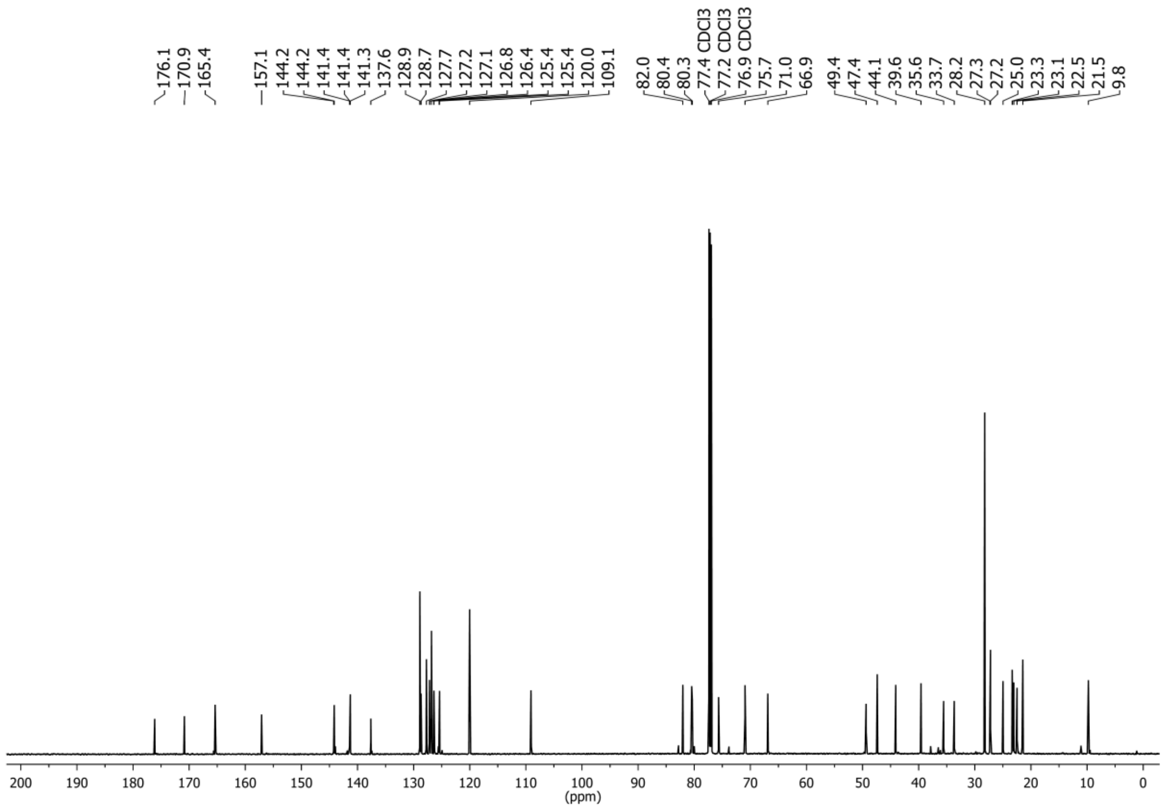

18: ^{1}H-NMR and ^{13}C-NMR

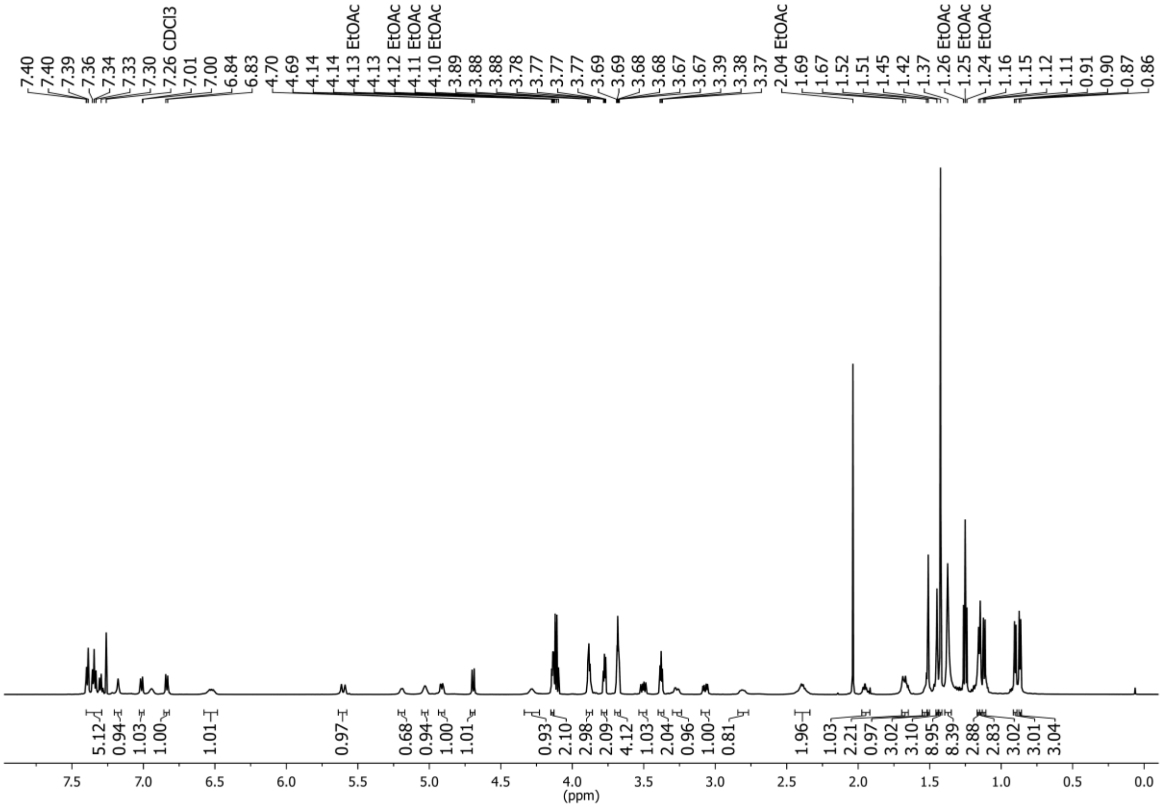

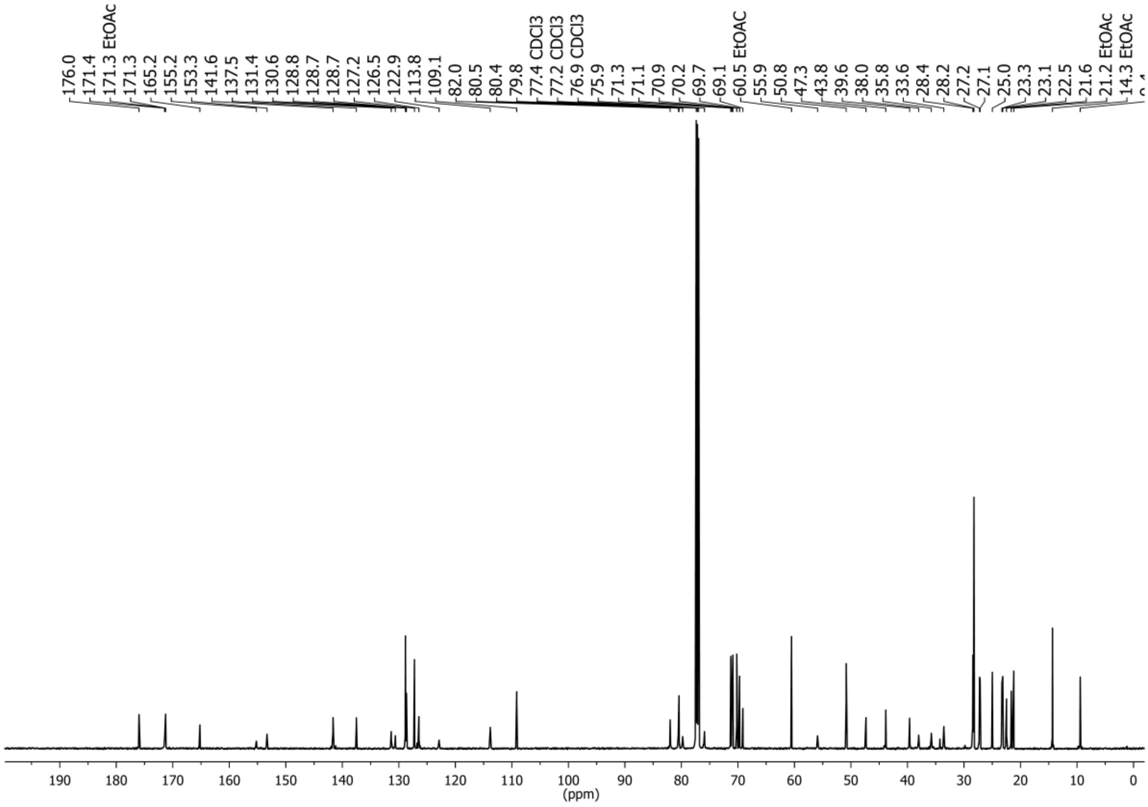

20: ^{1}H-NMR, ^{13}C-NMR and HPLC-MS

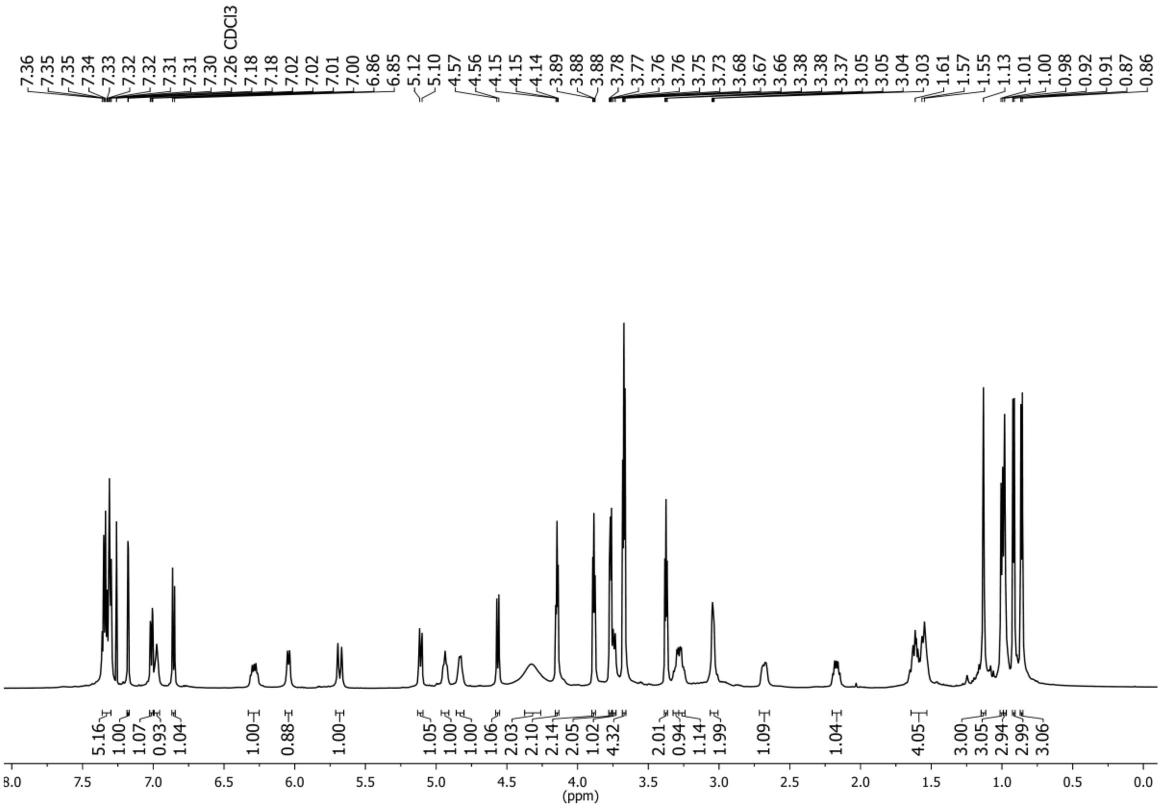

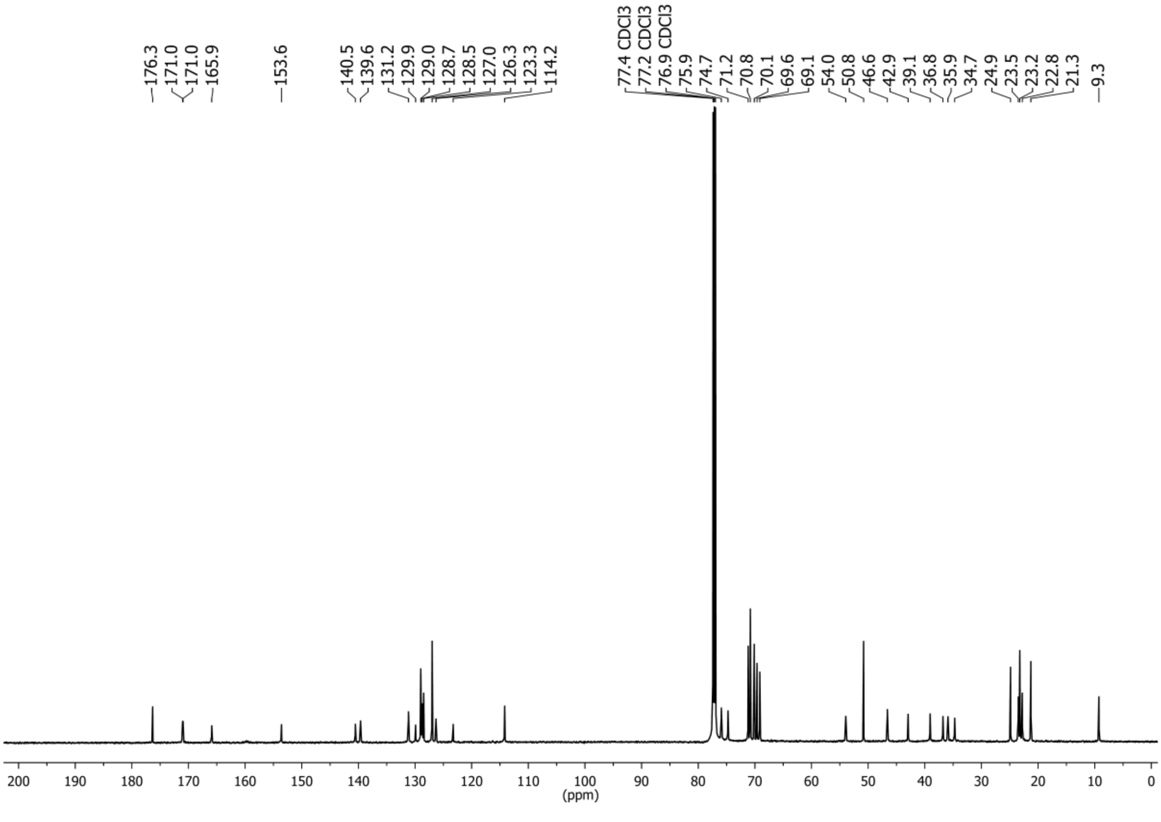

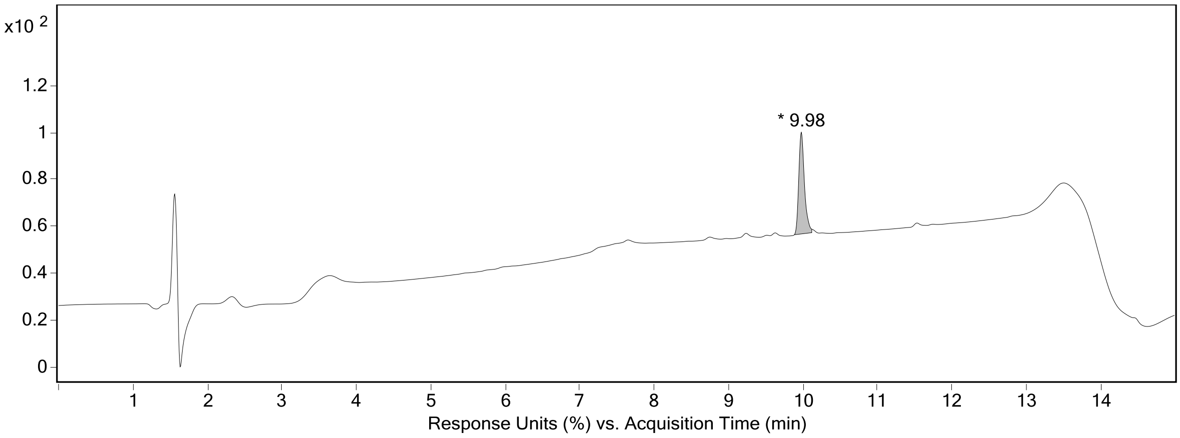
x10 2
* 9.98
Response Units (%) vs. Acquisition Time (min)

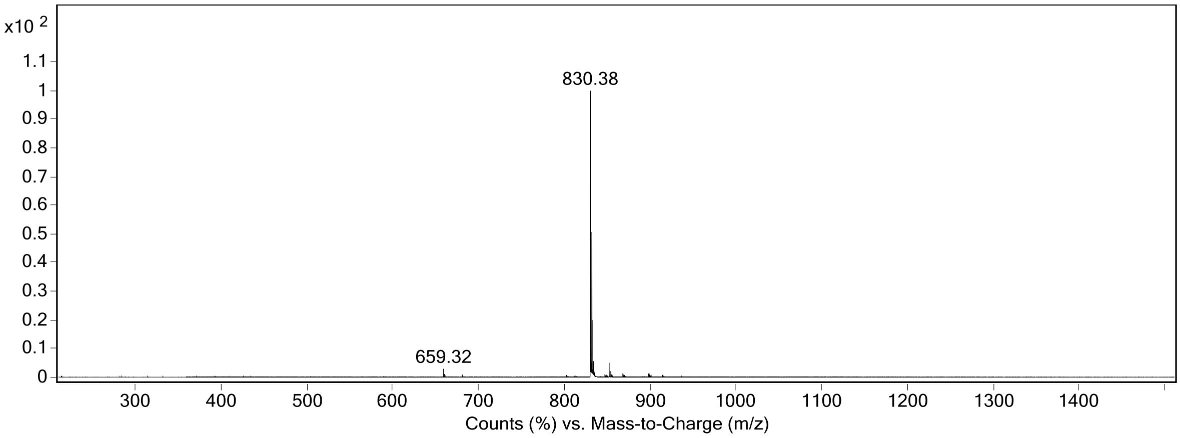
x10 2
830.38
659.32
Counts (%) vs. Mass-to-Charge (m/z)

22: ^{1}H-NMR, ^{13}C-NMR, HPLC-MS and HRMS

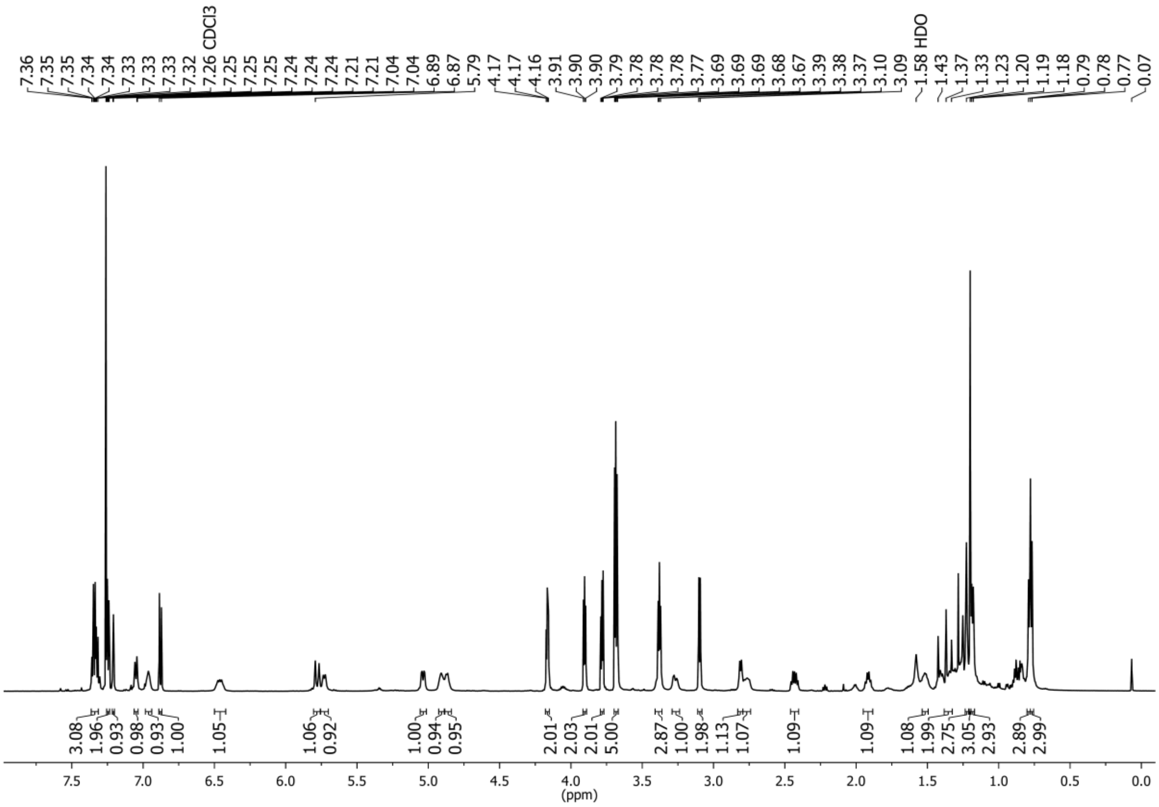

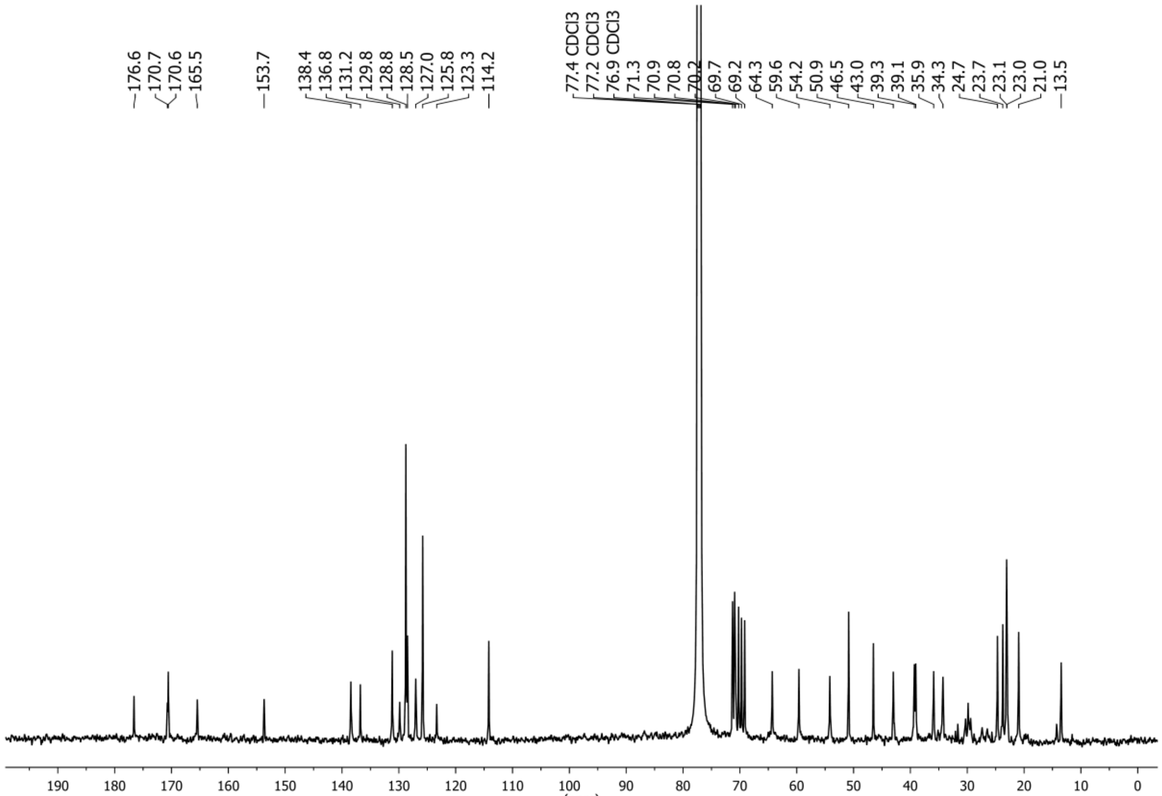

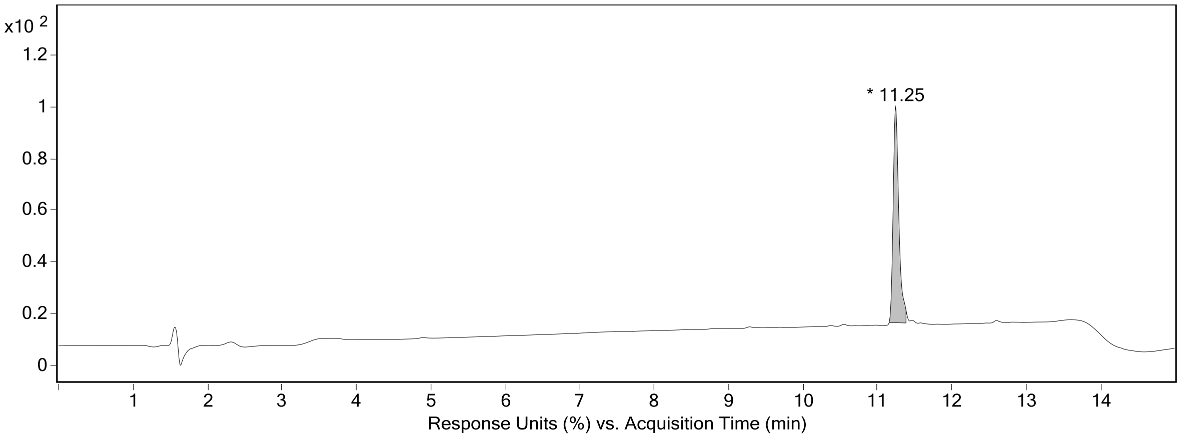
x10 2
* 11.25
Response Units (%) vs. Acquisition Time (min)

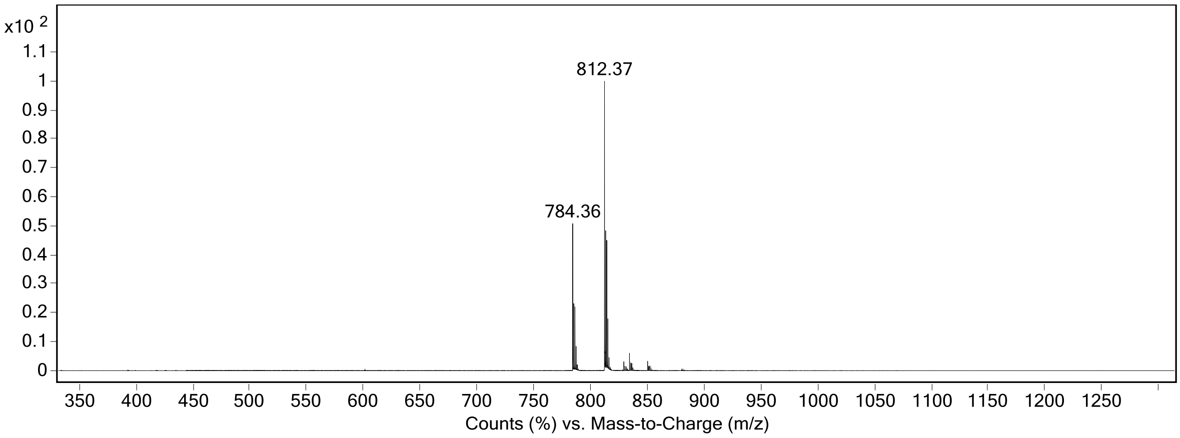
x10 2
812.37
784.36
Counts (%) vs. Mass-to-Charge (m/z)

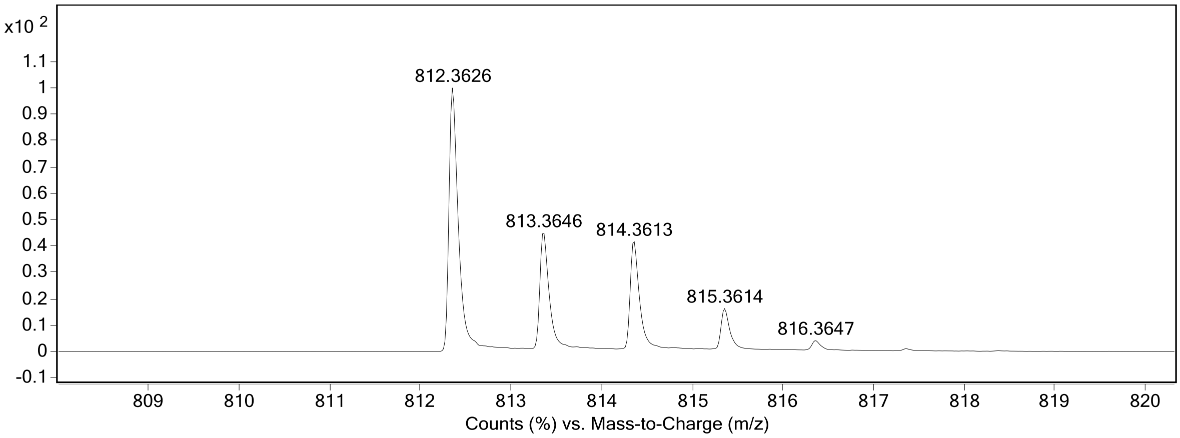
x10 2
812.3626
813.3646
814.3613
815.3614
816.3647
Counts (%) vs. Mass-to-Charge (m/z)

19: ^{1}H-NMR and ^{13}C-NMR

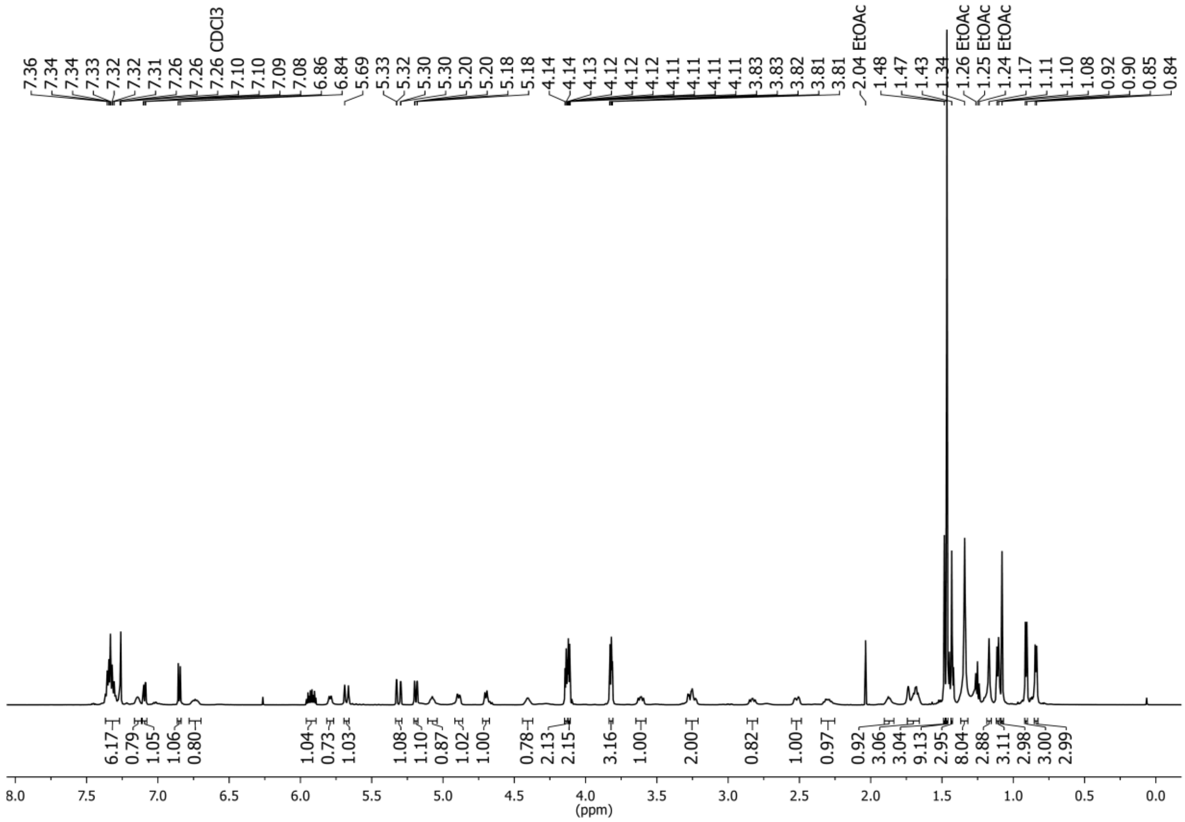

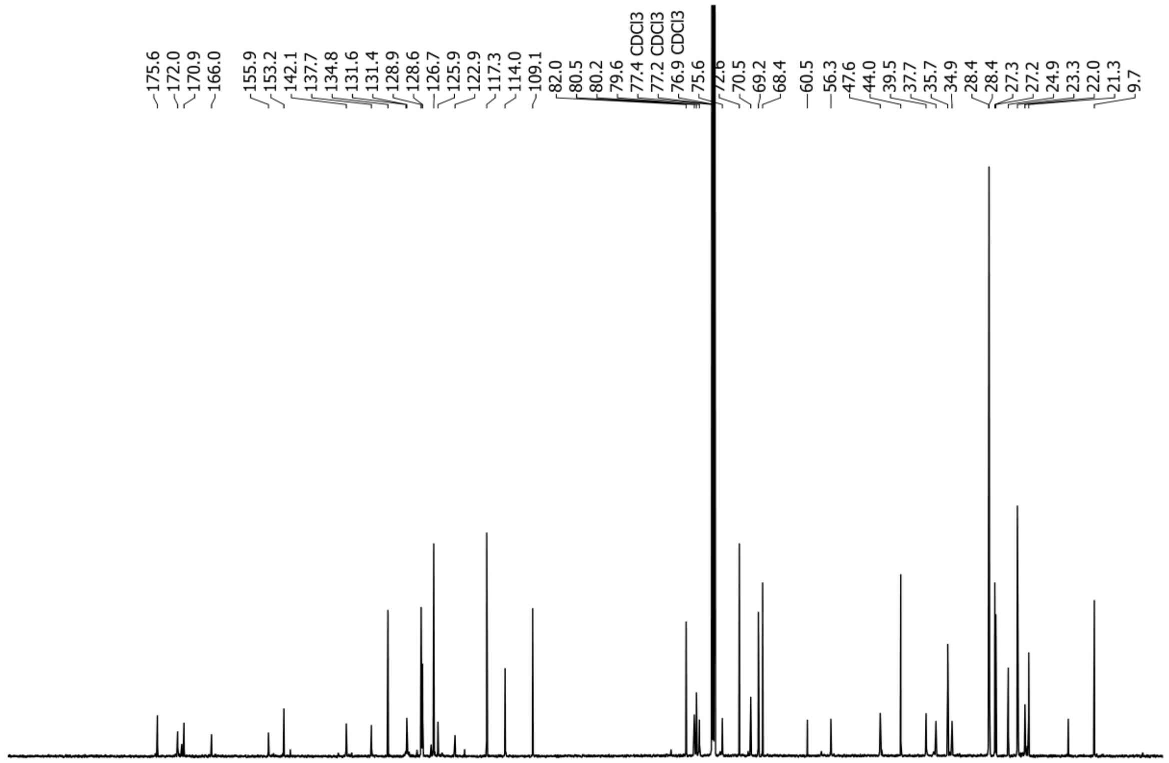

21: ^{1}H-NMR, ^{13}C-NMR and HPLC-MS

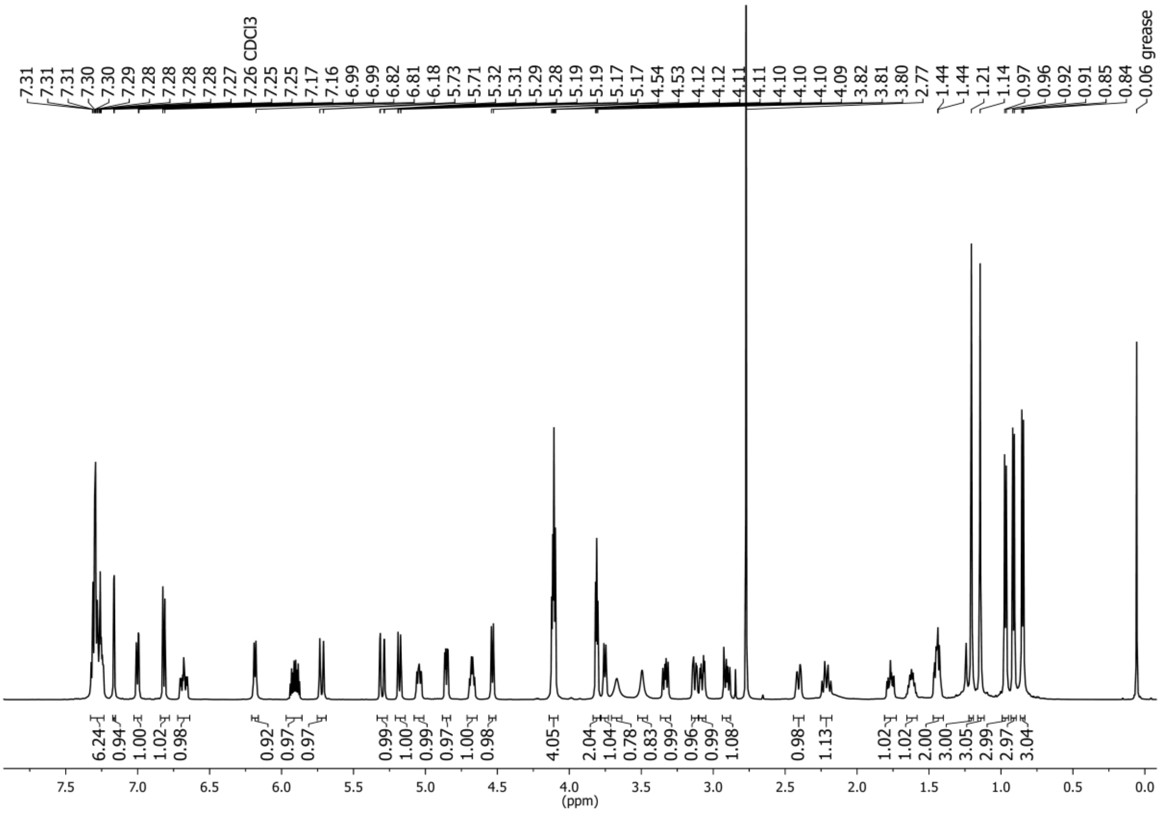

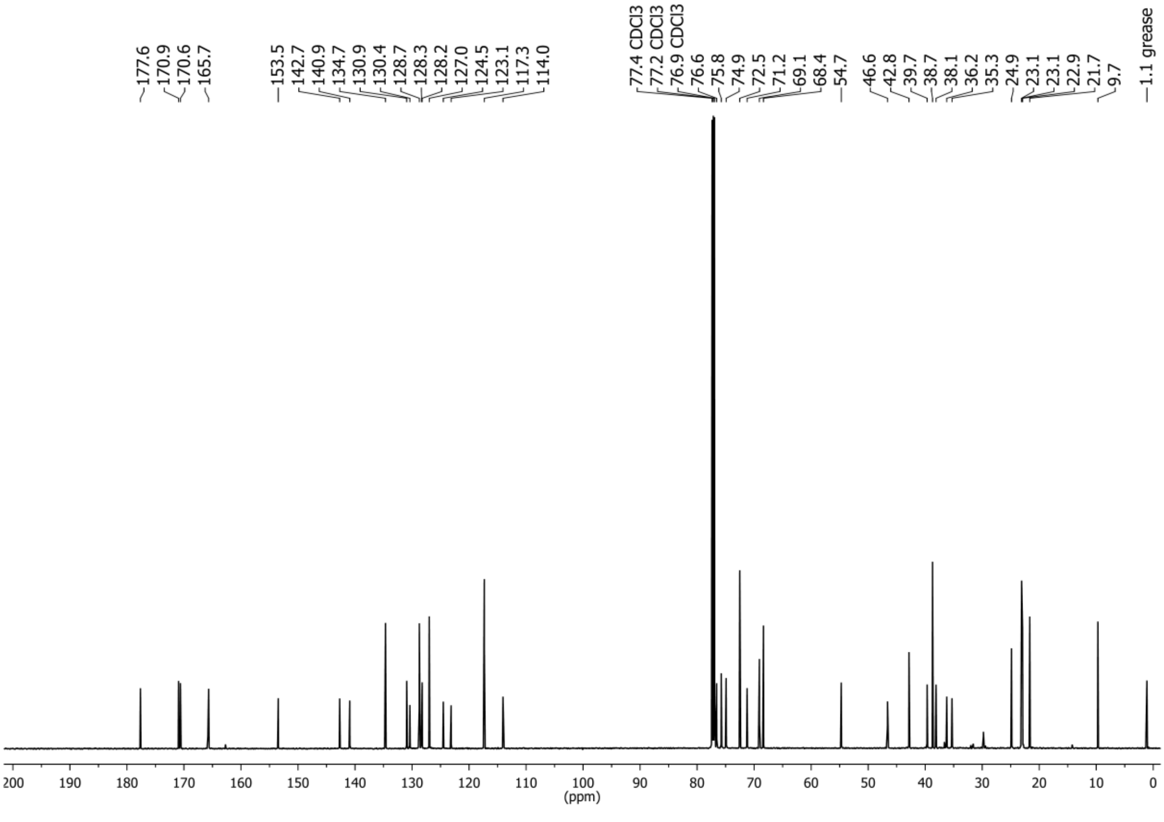

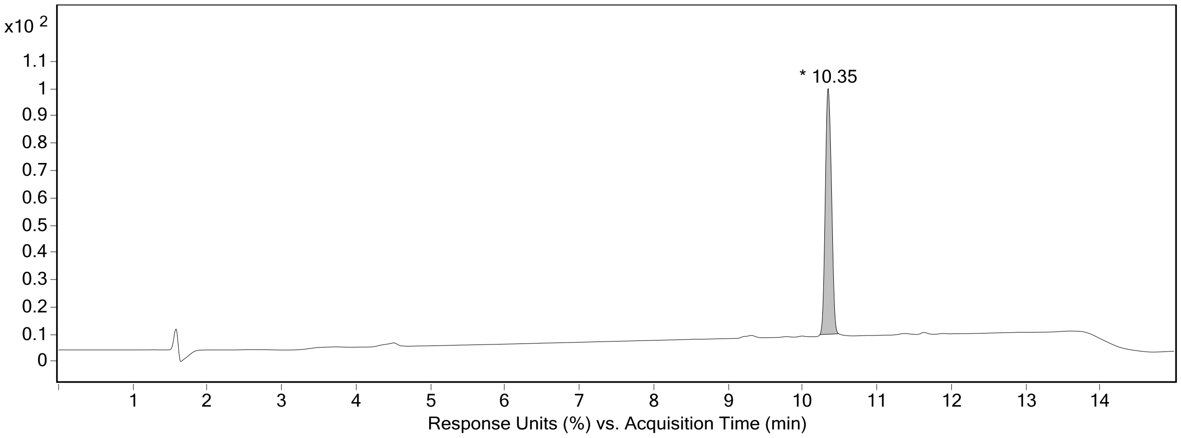
x10 2
* 10.35
Response Units (%) vs. Acquisition Time (min)

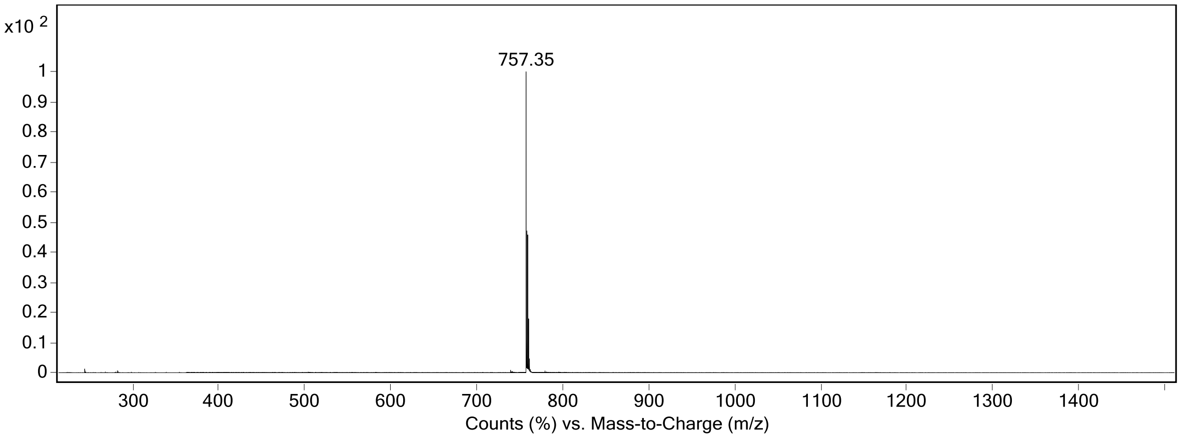
x10 2
757.35
Counts (%) vs. Mass-to-Charge (m/z)

23: ^{1}H-NMR, ^{13}C-NMR, HPLC-MS and HRMS

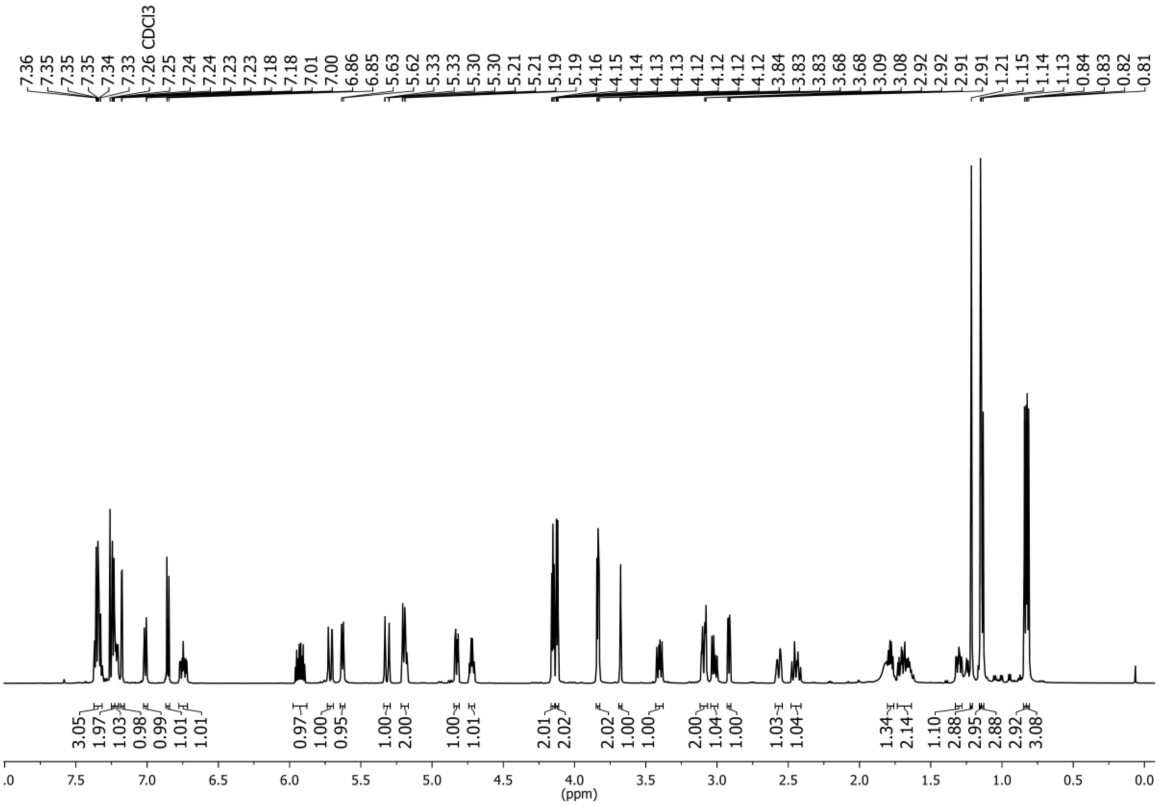

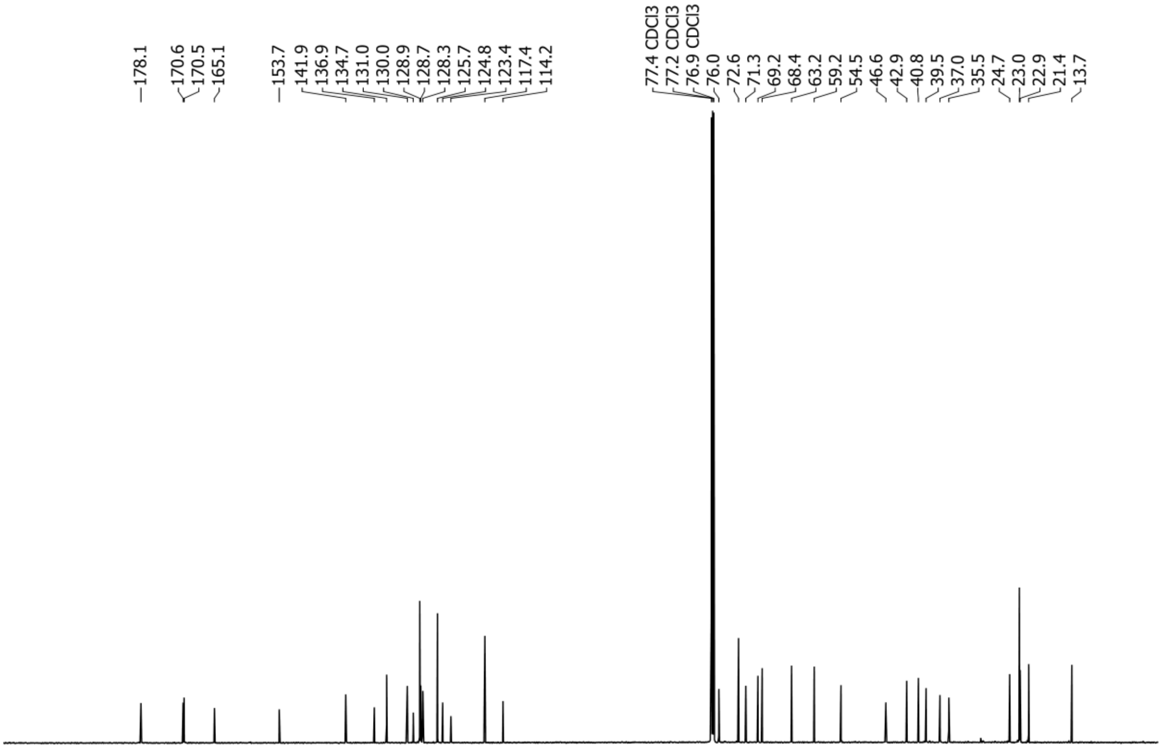

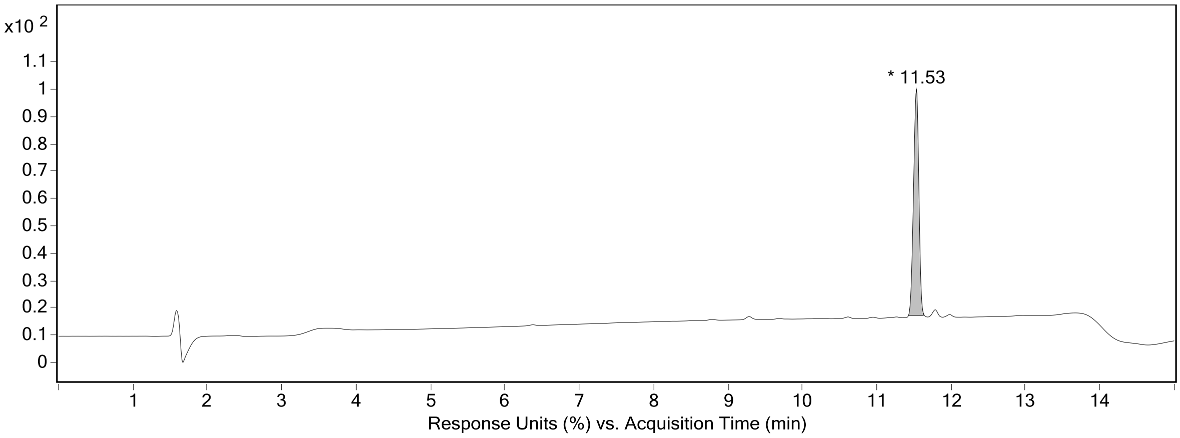
x10 2
* 11.53
Response Units (%) vs. Acquisition Time (min)

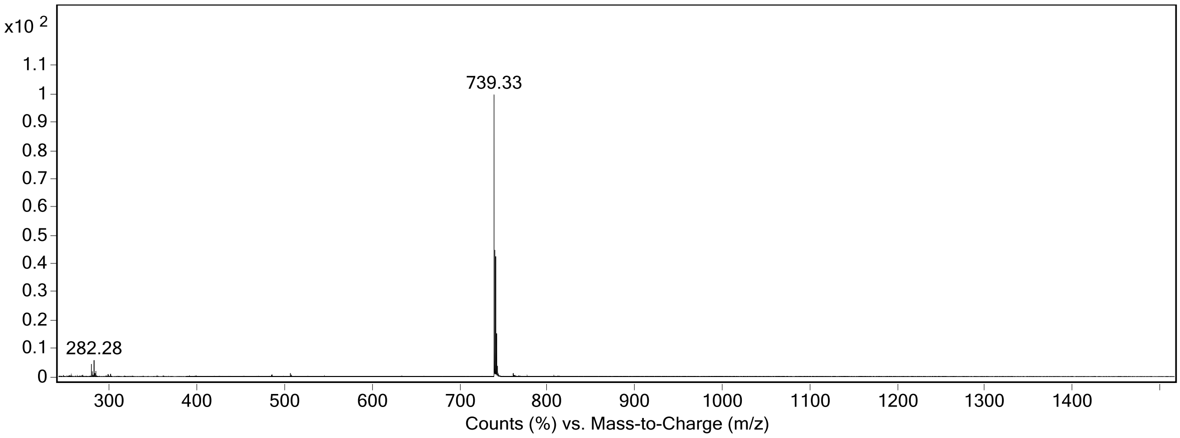
x10 2
739.33
282.28
Counts (%) vs. Mass-to-Charge (m/z)

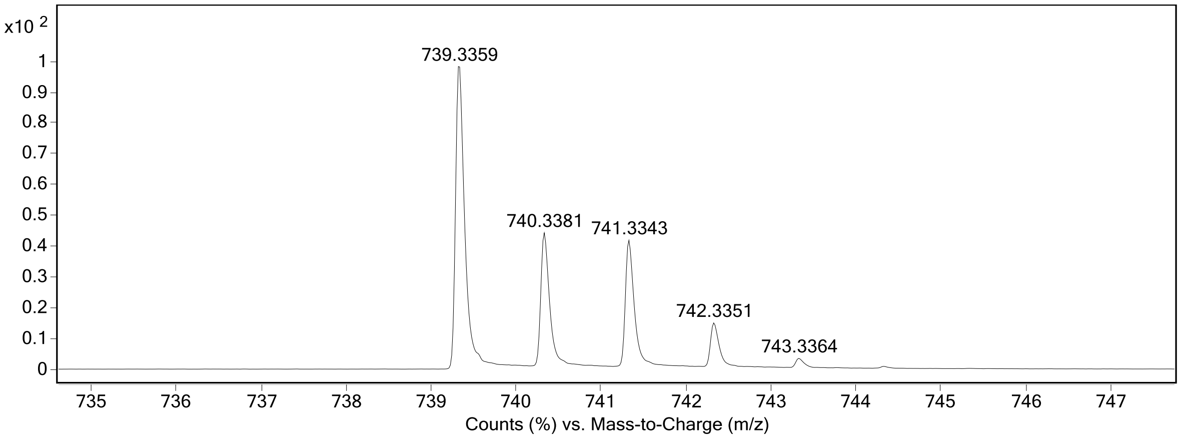
x10 2
739.3359
740.3381
741.3343
742.3351
743.3364
Counts (%) vs. Mass-to-Charge (m/z)

24: ^{1}H-NMR, HPLC-MS and HRMS

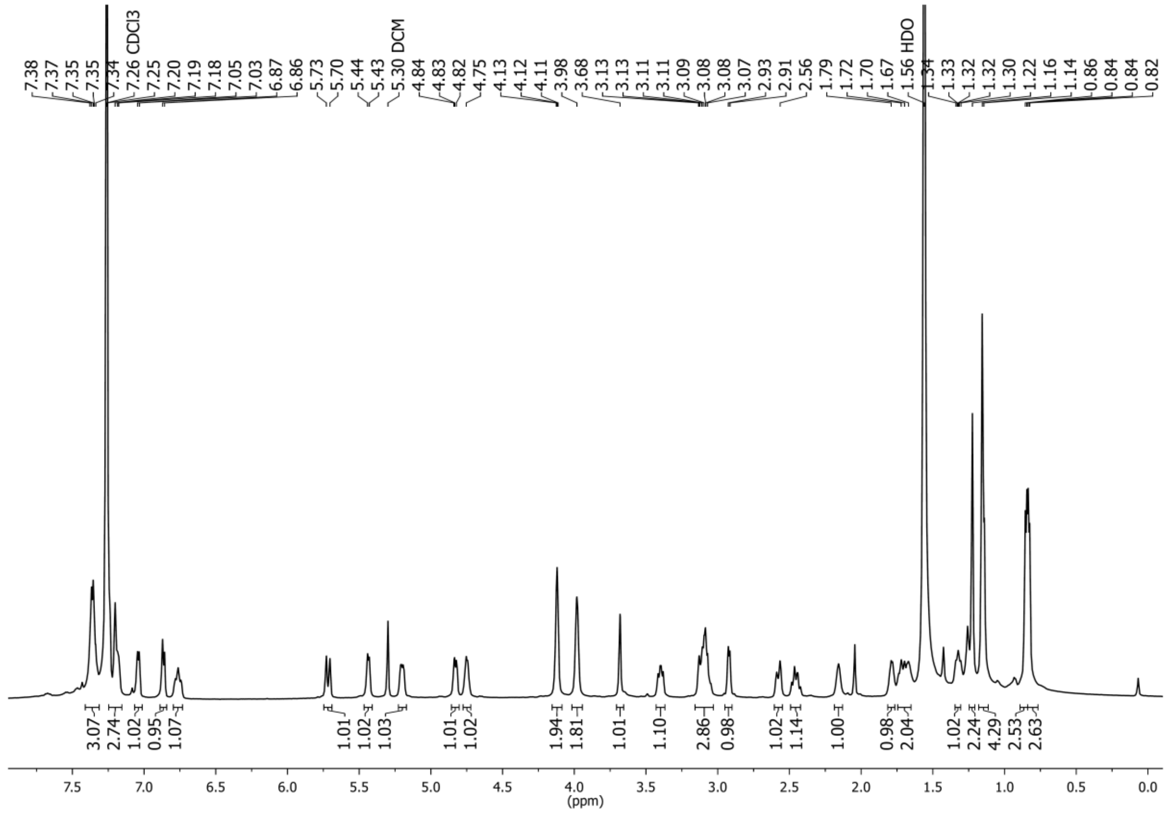

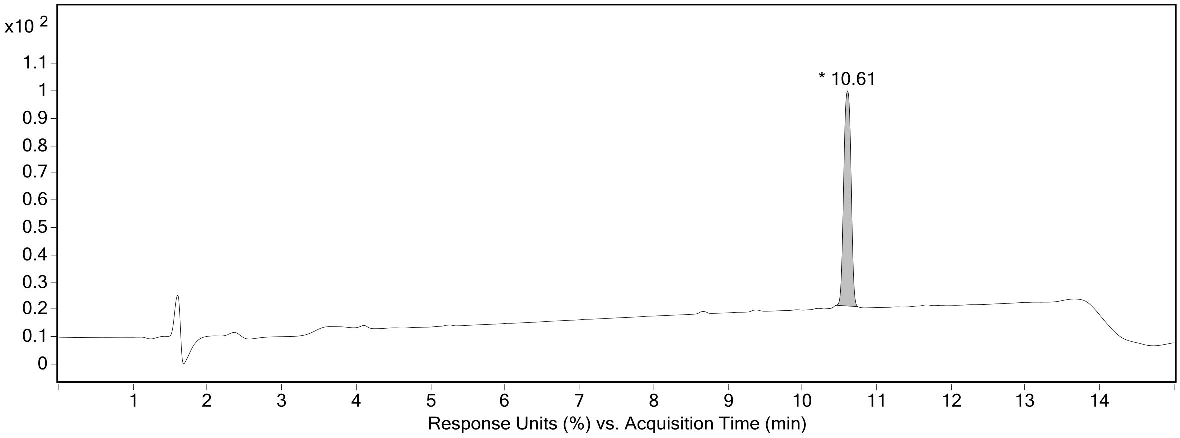

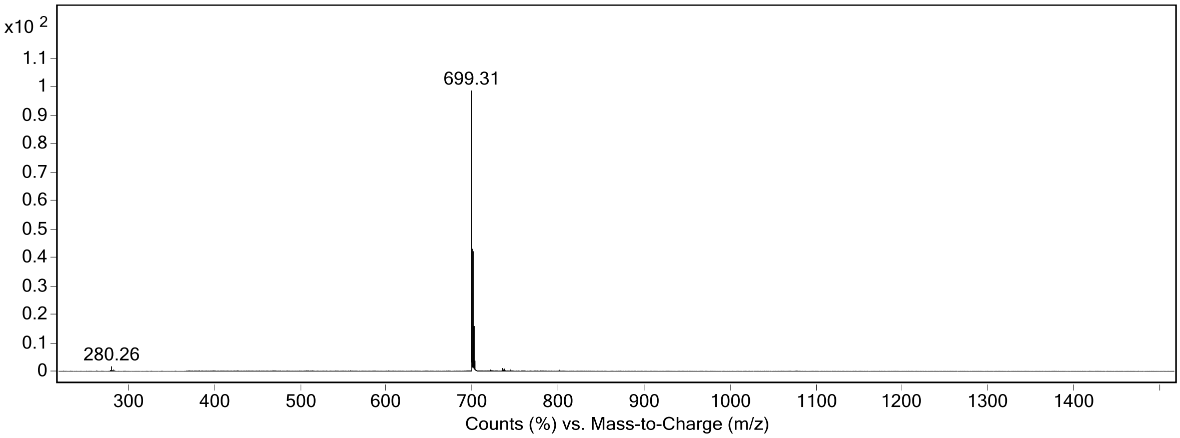

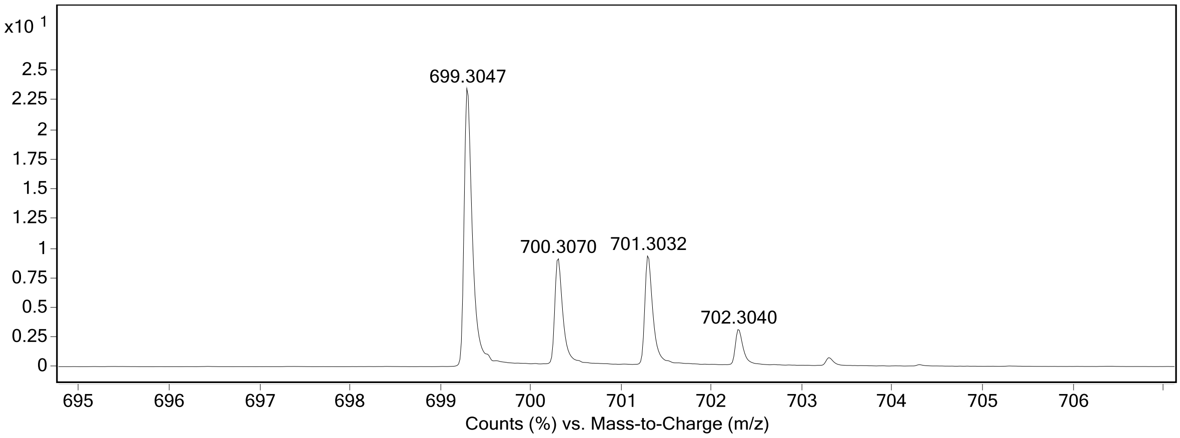

5) References

[1] G. M. Morris, D. S. Goodsell, R. S. Halliday, R. Huey, W. E. Hart, R. K. Belew, A. J. Olson, *J. Comput. Chem.* **1998**, *19*, 1639–1662.

[2] L. Börjesson, C. J. Welch, *Acta Chem. Scand.* **1991**, *45*, 621–626.

[3] S. Eissler, M. Nahrwold, B. Neumann, H.-G. Stammler, N. Sewald, *Org. Lett.* **2007**, *9*, 817–819.

[4] M. Nahrwold, C. Weiß, T. Bogner, F. Mertink, J. Conradi, B. Sammet, R. Palmisano, S. Royo Gracia, T. Preuße, N. Sewald, *J. Med. Chem.* **2013**, *56*, 1853–1864.
